# Fasudil induces anti-inflammatory transcriptomic changes and increased proliferation in human trisomy 21 neural progenitor cells

**DOI:** 10.1242/bio.062807

**Published:** 2026-09-08

**Authors:** Laura L. Baxter, Sarah E. Lee, Kevin A. Fuentes, Iman A. Mosley, Jonathan D. Raymond, Faycal Guedj, Di Zhou, Donna K. Slonim, Elliot J. Glotfelty, David Tweedie, Nigel H. Greig, Diana W. Bianchi

**Affiliations:** ^1^ Center for Precision Health Research, Prenatal Genomics and Therapy Section, National Human Genome Research Institute, National Institutes of Health, Bethesda, MD 20892, USA; ^2^ Department of Computer Science, Tufts University, Medford, MA 02155, USA; ^3^ Cellular Stress and Inflammation Section, Intramural Research Program, National Institute on Drug Abuse, National Institutes of Health, Baltimore, MD 21224, USA; ^4^ Drug Design & Development Section, Translational Gerontology Branch, Intramural Research Program, National Institute on Aging, National Institutes of Health, Baltimore, MD 21224, USA

**Keywords:** Down syndrome, Trisomy 21, Fasudil, Rho kinase inhibitor, Neural progenitor cells, Inflammatory pathways

## Abstract

Down syndrome (DS) results from trisomy for human chromosome 21 and is the most frequent genetic cause of intellectual disability. No effective treatments currently exist that improve neurodevelopment and cognition. Atypical brain development in individuals with DS is apparent before birth, which suggests that the optimal time to begin administration of therapies is prenatally. Human neural progenitor cell (NPC) cultures provide a tractable *in vitro* model system to examine the effects of trisomy 21 (T21) on neurodevelopment and to measure the effects of pharmacological interventions. Here, we report the results of preclinical studies evaluating 24 candidate therapies. RNA sequencing analyses found that euploid and T21 NPCs showed different transcriptomic responses to five candidate pharmacotherapies. The Rho-associated coiled-coil kinase inhibitor fasudil increased proliferation of T21 NPCs, reduced expression of inflammatory pathway genes in T21 NPCs, and reduced markers of inflammation in LPS-stimulated microglial model systems. These results demonstrate that fasudil can alter multiple T21-associated abnormalities in a beneficial manner, suggesting that fasudil warrants further study as a candidate prenatal pharmacotherapy for DS.

## Teaser

**Summary:** In trisomy 21 neural progenitor cells, fasudil increased growth and reduced expression of inflammatory pathway genes, indicating its potential as a candidate for Down syndrome prenatal therapy.

## INTRODUCTION

Down syndrome (DS) is caused by trisomy of human chromosome 21 [trisomy 21 (T21)] and is the most common genetic cause of intellectual disability. T21 is associated with atypical brain development that is apparent before birth. Magnetic resonance imaging (MRI) and transvaginal neurosonography in living fetuses with T21 have shown that structural alterations and regional brain hypoplasia are apparent in the developing fetal brain (Kitano et al., 2023; Pooh et al., 2025; Tarui et al., 2020; Yun et al., 2021). The early appearance of these neurodevelopmental abnormalities suggests that prenatal treatment may be required to counteract these effects. We and others hypothesize that safe and efficacious prenatal treatment will result in more typical fetal brain growth and development that could lead to improved learning, memory, and independent life skills for individuals with T21 (Guedj et al., 2014; Stagni and Bartesaghi, 2022; Stagni et al., 2015a).

Prenatal screening for DS provides a unique opportunity for fetal treatment for several reasons. Biochemical, cell-free DNA, and sonographic screening for T21 are part of routine prenatal care in most developed countries, allowing identification of high-risk fetuses during early, critical stages of brain development. Subsequent diagnostic testing via amniocentesis or chorionic villus sampling, followed by karyotype or chromosome microarray, would limit treatment to only those fetuses with confirmed T21. Furthermore, imaging measurements of T21-associated fetal brain changes have already provided baseline phenotypic data that could be used to measure the impact of antenatal therapy on brain growth and development (Kitano et al., 2023; Pooh et al., 2025; Tarui et al., 2020; Yun et al., 2021).

Given the limited availability of fetal brain tissue from individuals with T21, mouse models have previously been used in multiple studies to examine the effects of candidate therapeutics on neurodevelopment during embryonic and neonatal stages that correspond to the human fetal time period. Collectively, these prior efforts examined 15 prenatally and 12 neonatally administered molecules (21 molecules total) in the Ts65Dn, Dp(16)1Yey, and Ts1Cje mouse models of DS (López-Hidalgo et al., 2024; Stagni and Bartesaghi, 2022). These studies demonstrated that central nervous system (CNS) development and subsequent cognitive abilities can be improved by pharmacological intervention early in development, with the most significant results arising from prenatal administration (Stagni and Bartesaghi, 2022).

The candidate therapeutics analyzed in prior mouse studies were chosen using a variety of criteria. These included targeting CNS pathways known to be altered in humans with T21 or mouse models of DS, such as the serotonin reuptake inhibitor fluoxetine (Guidi et al., 2014) or the tropomyosin-related kinase receptor B agonist 7,8-dihydroxyflavone (Giacomini et al., 2019; Stagni et al., 2017, 2021; Valenti et al., 2021), or inhibiting proteins encoded by trisomic genes such as the antioxidant (−)-epigallocatechin-3-gallate (EGCG), which inhibits DYRK1A (McElyea et al., 2016; Souchet et al., 2019; Stagni et al., 2016; Tielemans et al., 2025). In addition, our laboratory used a transcriptomics approach to identify novel therapeutic candidate molecules (Guedj et al., 2016). Dysregulated gene sets from nine cell and tissue types from human T21 sources and mouse models of DS were used to query the Connectivity Map database (Lamb et al., 2006) to discover molecules predicted to improve dysregulated gene expression. This identified the naturally occurring flavone apigenin (4′,5,7-trihydroxyflavone) as a candidate (Guedj et al., 2016). Subsequent studies (Guedj et al., 2020) showed that apigenin reduced oxidative stress and enhanced antioxidant defense responses in T21 amniocytes. In addition, administration of apigenin to pregnant Ts1Cje dams followed by administration to offspring over their lifetimes reduced inflammatory markers, improved neonatal developmental milestones, and improved exploratory behavior and hippocampal long-term memory in adult male mice (Guedj et al., 2020). These results showed that a transcriptomic approach could successfully identify new therapeutic molecules that improve trisomy-associated neurodevelopmental alterations.

Although many candidate molecules showed promise in mouse model studies, similarly beneficial effects were not seen in clinical trials of individuals with DS (Rueda et al., 2020a). This lack of translation may be due to differences in murine and human neurodevelopment, resulting in different phenotypic effects of trisomy. It may also be attributable to incomplete modeling of trisomy: the Ts65Dn, Dp(16)1Yey, and Ts1Cje mouse models used for these studies are trisomic only for a subset of genes that are orthologous to human chromosome 21 (*Hsa*21). Furthermore, Ts65Dn and Ts1Cje contain aneuploid regions that are not syntenic to *Hsa*21, which could influence therapeutic responses (Davisson et al., 1990; Duchon et al., 2011; Guedj et al., 2023; Reeves et al., 1995; Sago et al., 1998). Overall, these inconsistent results suggest that preclinical models need to be improved to better predict the effects of candidate therapeutics.

The recent creation of human induced pluripotent stem cells (iPSCs) from T21 somatic cells has provided a valuable alternative to mouse models for studying neurodevelopment in DS. These cells are fully trisomic for *Hsa*21 and can be differentiated into a variety of neuronal cell types (Klein and Haydar, 2022; Russo et al., 2024; Watson and Meharena, 2023). We previously created a panel of age- and sex-matched euploid (Eup) and T21 iPSCs and differentiated these iPSCs into neural progenitor cells (NPCs) (Lee et al., 2025). Characterization of these NPCs showed that T21 is associated with genome-wide disruption of the transcriptome, reduced growth, increased oxidative stress, and inter-individual variability in gene and protein expression. NPCs are relevant to T21 neurodevelopmental phenotypes because prenatal brain hypotrophy is associated with fewer proliferating NPCs and reduced neurogenesis (Contestabile et al., 2007; Guidi et al., 2008, 2018; Lu et al., 2012; Stagni et al., 2018, 2019, 2020). A previous study showed that the senolytics dasatinib and quercetin together reduced T21-associated cellular phenotypes in NPCs (Meharena et al., 2022), highlighting the utility of NPCs as an *in vitro* model system to test pharmacotherapies.

Here, we describe preclinical studies using our panel of T21 NPCs to evaluate candidate therapeutic molecules. Molecules were chosen for analysis based upon previously published literature that suggested their therapeutic potential or via a transcriptomic approach in which the Library of Integrated Network-Based Cellular Signatures (LINCS) was queried for molecules predicted to reverse T21 NPC gene expression signatures and thus partially restore gene expression to Eup levels (Keenan et al., 2018; Sapashnik et al., 2023). Therapeutic endpoints included proliferation of NPCs, normalization of differentially expressed genes (DEGs) and pathways in T21 NPCs, and reduction of lipopolysaccharide (LPS)-induced inflammation in mouse macrophages and microglia. These studies found that T21 and Eup cells do not show identical transcriptomic responses to molecules. In addition, these studies demonstrated that the Rho-associated coiled-coil kinase (ROCK) inhibitor fasudil increased proliferation in T21 NPCs, reduced expression of inflammatory pathways in T21 NPCs, and showed anti-inflammatory properties in Eup microglia, suggesting that fasudil shows potential for prenatal pharmacotherapy for DS.

## RESULTS

### Selection of candidate therapeutic molecules

**Fig. 1.**
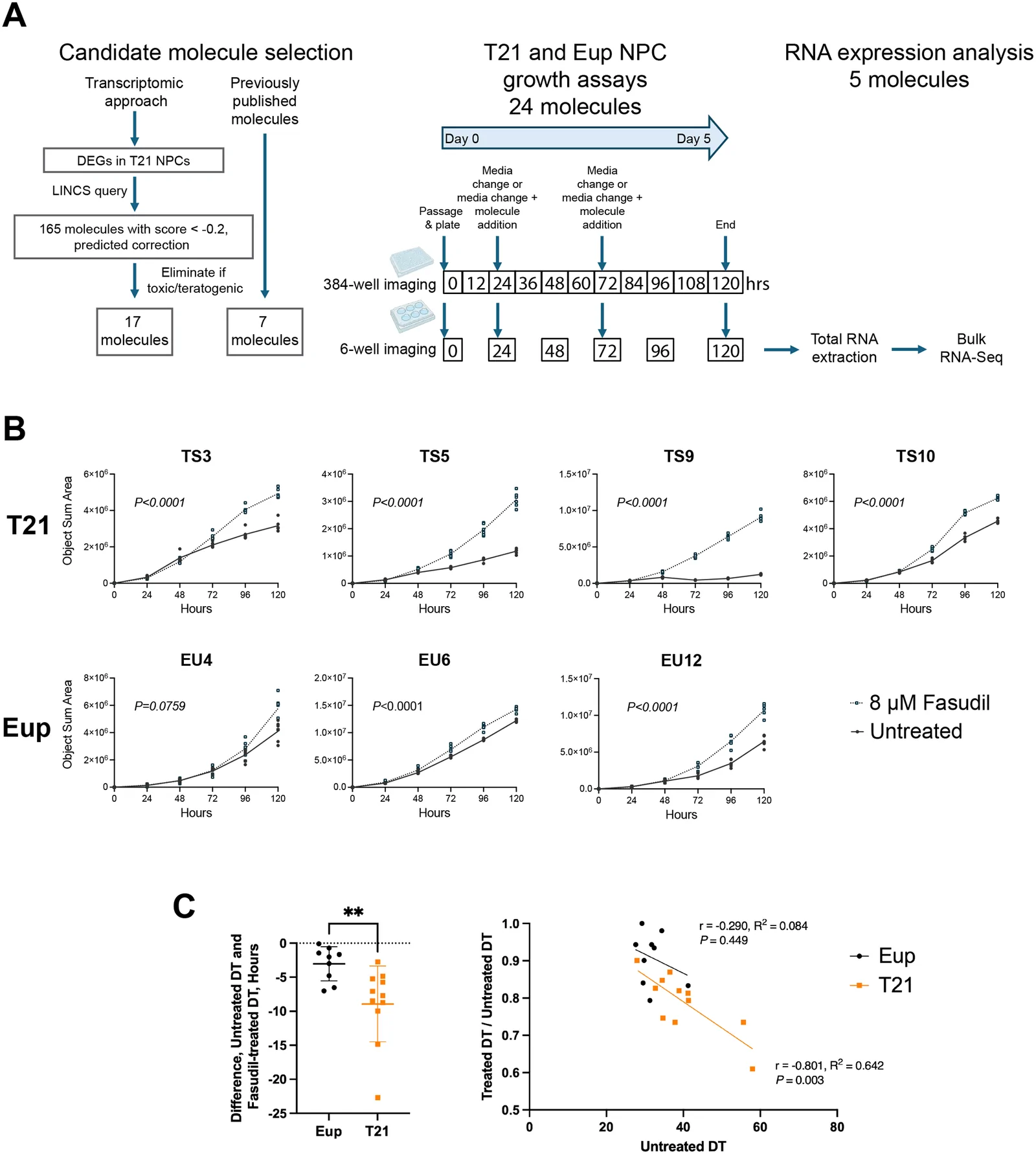
**Analyses of a panel of candidate therapeutics showed that fasudil improved T21 NPC proliferation.** (A) Experimental design. Twenty-four candidate molecules were selected from a transcriptomic approach, using DEGs in T21 NPCs to query LINCS, or from previously published results suggesting therapeutic benefits. Molecules were screened for cell proliferation effects in a 384-well plate format followed by a six-well plate format by quantitative live-cell imaging every 12 or 24 h, respectively. Two to four technical replicates were performed for 384-well-format experiments, with four identical wells per condition in each replicate. Two to three replicates were performed for six-well-format experiments, with six identical wells per condition in each replicate. Bulk RNA-seq analysis was performed on RNA collected from Eup and T21 NPCs treated with five molecules after 120 h. (B) Increased growth occurred following 8 µM fasudil treatment in all T21 lines (*N*=4) and in two out of three Eup lines (*N*=3). Each graph shows a single, representative experiment for one cell line in the six-well-format experiments (*N*=3 per line), with six replicate wells shown for each timepoint. Open squares=fasudil treated, closed circles=untreated. *P*-values are from two-way ANOVA. Data for all replicates are shown in Table S1b-d and Fig. S2. T21 and Eup lines are named using the prefixes TS and EU, respectively, and these lines are described in Table S2. (C) Greater reductions in DT occurred in T21 NPCs (graph, left). Mean DT changes after fasudil treatment=−8.92 h (s.d.=5.6) for T21 and −3.02 h (s.d.=2.5) for Eup (*P=*0.0016, Mann–Whitney two-tailed test). Fasudil-treated DT normalized to untreated DT showed greater effects in T21 NPCs (graph, right). The ratio of treated/untreated DT was significantly correlated with untreated DT in T21 lines, consistent with fasudil showing greater effects in slower-growing lines, while Eup NPCs showed no correlation. *N*=11 for T21, *N*=9 for Eup; each point shows DT for one experiment for a single NPC line (data in Table S1c). Black, Eup lines; orange, T21 lines. **P≤0.01.

Extending upon our previous transcriptomics approach to identify novel therapeutic candidate molecules (Guedj et al., 2016), we used a set of DEGs in T21 NPCs relative to Eup NPCs (Lee et al., 2025) to query LINCS (https://lincsproject.org/). LINCS is an NIH-funded, publicly available data repository of information on the cellular responses of over 70 human cell types to various genetic, disease, or chemical perturbations, including transcriptional responses to over 20,000 bioactive small molecules (Keenan et al., 2018). Submission of gene signatures for the T21 DEGs (DEGs plus expression changes for each gene) to LINCS identified 165 molecules with weighted connectivity scores <−0.2 in NPCs (Subramanian et al., 2017) (Fig. 1A). Negative scores indicate reversal of the gene signature query; therefore, these 165 molecules were predicted to correct gene expression in T21 NPCs toward Eup levels.

We used a translational research focus to prioritize the 165 LINCS-predicted molecules to increase the likelihood that promising candidate therapies would have greater potential for practical clinical applications. PubMed searches were performed on the 165 LINCS-predicted molecules to identify those with teratogenicity or adverse pregnancy effects. Seventeen of the molecules with no reported teratogenicity or contraindications in pregnancy were chosen for further examination using NPC cultures (Fig. 1A; Table S1a). Of note, previous studies on seven of these transcriptomically selected molecules showed beneficial effects in preclinical models of T21, neurodevelopment, neurodegeneration, or oxidative stress. These included the fatty acids alpha linolenic acid and eicosapentaenoic acid (García-Cerro et al., 2020; Martinez-Cue and Bartesaghi, 2022; Zmijewski et al., 2015), the PGC-1α activator metformin (Izzo et al., 2017), the flavonoids naringenin and nobiletin (Ghasemi-Tarie et al., 2022; Jahan et al., 2024; Nakajima and Ohizumi, 2019; Nouri et al., 2019; Xiong et al., 2023), the peroxisome proliferator-activated receptor-γ agonist pioglitazone (Mollo et al., 2019), and the lipid sphingosine (Hwang et al., 2019; Tang et al., 2017). No information on therapeutic effects in preclinical models of T21 was found in PubMed for the remaining ten molecules identified by the LINCS query (chloroquine, cilnidipine, geranylgeraniol, glycodeoxycholic acid, imperatorin, isoxsuprine, pinocembrin, tangeretin, trapidil, and vitexin).

Alongside the candidate therapeutic molecules identified by transcriptomic analysis, seven molecules with previously published data suggesting potential therapeutic benefits in T21 model systems or individuals with DS were also chosen for analysis in NPCs (Fig. 1A; Table S1a). These molecules were apigenin, which we previously identified using a transcriptional approach (Guedj et al., 2020), the flavonoid 7,8-dihydroxyflavone (Giacomini et al., 2019; Stagni et al., 2017, 2021; Valenti et al., 2021), choline chloride (Alldred et al., 2023, 2025; Gautier et al., 2023, 2024; Moon et al., 2010; Powers et al., 2017, 2021; Velazquez et al., 2013), the antioxidants curcumin (Rueda et al., 2020b) and EGCG (Blazek et al., 2015; Catuara-Solarz et al., 2015; de la Torre et al., 2016, 2014; McElyea et al., 2016; Souchet et al., 2019; Stagni et al., 2016; Starbuck et al., 2021; Tielemans et al., 2025), the ROCK inhibitor fasudil (LeBlanc-Straceski et al., 2022 preprint; López-Hidalgo et al., 2024), and the selective serotonin reuptake inhibitor fluoxetine (Bianchi et al., 2010; Guidi et al., 2014, 2013; Heinen et al., 2012; Stagni et al., 2015b, 2013).

### Analysis of cell proliferation in response to candidate therapeutic treatment

T21 NPCs show reduced proliferation *in vitro*, and immunostaining of T21 fetal brains suggests that fewer proliferating cells are present *in vivo* (Contestabile et al., 2007; Guidi et al., 2008, 2018; Lee et al., 2025; Lu et al., 2012; Stagni et al., 2018, 2019, 2020); thus, we examined the effects of candidate pharmacotherapies on NPC growth. These experiments used a panel of NPCs derived from iPSC lines that were previously generated from fibroblasts of unrelated Eup individuals and individuals with T21 (Lee et al., 2025). First, 17 iPSC lines (nine Eup and eight T21, Table S2) were stably transduced with Nuclight Red, a nuclear-restricted, non-integrating, lentivirus-derived fluorescent vector. Transduced iPSCs were then differentiated into NPCs that retained Nuclight Red expression, which permitted automated counting of live NPCs for growth measurement.

T21 NPC lines exhibited variable recovery from passaging when supplemented with the commonly used ROCK inhibitor Y-27632. To address this issue, we tested a protocol during neural induction from iPSCs that reportedly improved survival during passaging by supplementation of media with the small molecule ‘CEPT’ cocktail of chroman 1 (a ROCK inhibitor), emricasan, polyamines, and trans-ISRIB (Chen et al., 2021). Administration of CEPT during passaging of three Eup and three T21 lines led to greater cell survival following passaging in both genotypes that reduced variability among replicates and permitted more consistent evaluation of cellular proliferation (Fig. S1). Therefore, CEPT supplementation was used for passaging throughout all NPC analyses.

A 384-well plate format was used to examine the effects of candidate pharmacotherapies on NPC growth (Fig. 1A). A panel of Eup and T21 Nuclight-Red-transduced NPC lines (four to five Eup and five to six T21 lines per molecule; Table S2) was tested for each molecule. Eup and T21 NPCs were cultured for 120 h in the presence or absence of candidate molecules at two or three concentrations (based on previous *in vitro* dose-range-finding applications), and quantitative live-cell imaging was performed every 12 h to generate growth curves. Each NPC line was evaluated in quadruplicate wells for each molecule, and growth assays were repeated at least once for every molecule (two to three replicates for each molecule per line). Eight molecules caused decreased growth and increased doubling time (DT) in Eup and T21 NPCs relative to untreated NPCs, indicating potential toxic effects (Table S1b). The remaining molecules showed no change in DT or variable, inconsistent effects on DT. Of note, 8 µM fasudil showed improved growth and reduced DT in 18% of the experimental replicates across all T21 cell lines, the most of any molecules tested in the 384-well plate format (Table S1b).

### Fasudil consistently increases cell growth in T21 NPCs

Additional analyses of the effects of candidate therapeutics on cell proliferation were performed on NPCs in six-well plates (Fig. 1A) to determine if similar growth effects were seen in large-volume culture conditions and to generate samples for RNA collection. Five molecules were used: two previously identified candidate therapeutics, fasudil (8 µM) and EGCG (8 µM), and three transcriptionally identified molecules, apigenin (4 µM), isoxsuprine (1 µM), and sphingosine (2 µM). EGCG, apigenin, and isoxsuprine did not consistently change growth (Table S1c). Sphingosine showed no consistent effects on growth in T21 NPCs but consistently increased DT (thus slowing growth) in two Eup lines (Table S1c). Interestingly, fasudil reduced DT (thus improving growth) in four T21 NPC lines, and this effect was reproducible across all replicate experiments (Table S1c). Two-way analyses of variance (ANOVAs) of growth curves confirmed significantly increased growth in all fasudil-treated T21 (T21Fas) NPCs across all replicate experiments (two to three replicates for each molecule per line, Fig. 1B; Fig. S2, Table S1d). In Eup lines, fasudil showed less consistent effects on growth in comparison to T21 lines. Fasudil consistently reduced DT in Eup line EU12, but Eup lines EU4 and EU6 showed reduced DT in only one or two experiments (Table S1c). In two-way ANOVAs of Eup growth curves, EU4 showed no change, EU6 showed consistently increased growth across all three experiments, and EU12 showed increased growth in two out of three experiments (Fig. 1B; Table S1d).

Several T21 lines showed slower growing times/longer DTs than Eup lines in untreated conditions (Table S1c). Therefore, we wanted to determine if fasudil caused stronger growth effects in T21 NPCs. Indeed, greater reductions in DT were seen in T21 NPCs in comparison to Eup NPCs in the six-well format cultures, with mean DT changes after fasudil treatment of −8.92 h (s.d.=5.6) for T21 and −3.02 h (s.d.=2.5) for Eup (*P*=0.0016, Mann–Whitney two-tailed test, Fig. 1C). T21 lines showed greater variability in DTs, and the largest change in DT was seen in the slowest-growing T21 line [line TS5, mean DT change=−14.27 h (s.d.=8.7)]. The ratio of treated/untreated DT was correlated with untreated DT in T21 lines (*r*=−0.801, *R*^2^=0.642, *P*=0.003, Fig. 1C), consistent with fasudil showing greater effects in slower-growing lines. In contrast, no correlation was present between the ratio of treated/untreated DT and untreated DT in Eup NPCs (Fig. 1C).

### Anti-inflammatory properties of fasudil, apigenin, and isoxsuprine

Candidate pharmacotherapies with anti-inflammatory properties are of interest because upregulated interferon and inflammatory pathways contribute to multiple phenotypes in mouse models of DS and individuals with T21 (Chi et al., 2023; Galbraith et al., 2023; Rachubinski et al., 2024; Sullivan et al., 2017, 2016; Tuttle et al., 2020; Waugh et al., 2023). Microglia are resident macrophages in the brain that function as the brain's innate immune system. As a source of inflammatory signaling, microglia play integral roles in shaping neural circuits and maintaining brain homeostasis, which is crucial during development of the fetal brain (Anderson et al., 2025; Lawrence et al., 2024). *In vitro* microglia/macrophage cultures have previously been used to recapitulate *in vivo* and primary microglial inflammatory phenotypes across many drug classes (Glotfelty et al., 2023; Hsueh et al., 2025; Kopp et al., 2024; Lecca et al., 2022; Li et al., 2021). These culture systems were applied to the candidate molecules fasudil, EGCG, apigenin, isoxsuprine, and sphingosine using the classical LPS challenge model, in which LPS induces an inflammatory response that is mediated by Toll-like receptor 4.

LPS challenge was first applied to the RAW 264.7 cell line, a mouse macrophage cell line reported to have parallel features to blood-induced microglia (Cheon et al., 2017; Mendiola et al., 2023). Cellular viability evaluation at 1, 10, and 100 µM concentrations showed that EGCG, fasudil, apigenin, and sphingosine were toxic to RAW 264.7 cells at 100 µM concentrations, reducing the levels of cell viability to 80±2%, 33±1%, 55±1%, and <1% compared to the control group (vehicle), respectively. Consequently, the highest compound concentration assessed in subsequent studies was 30 µM.

**Fig. 2.**
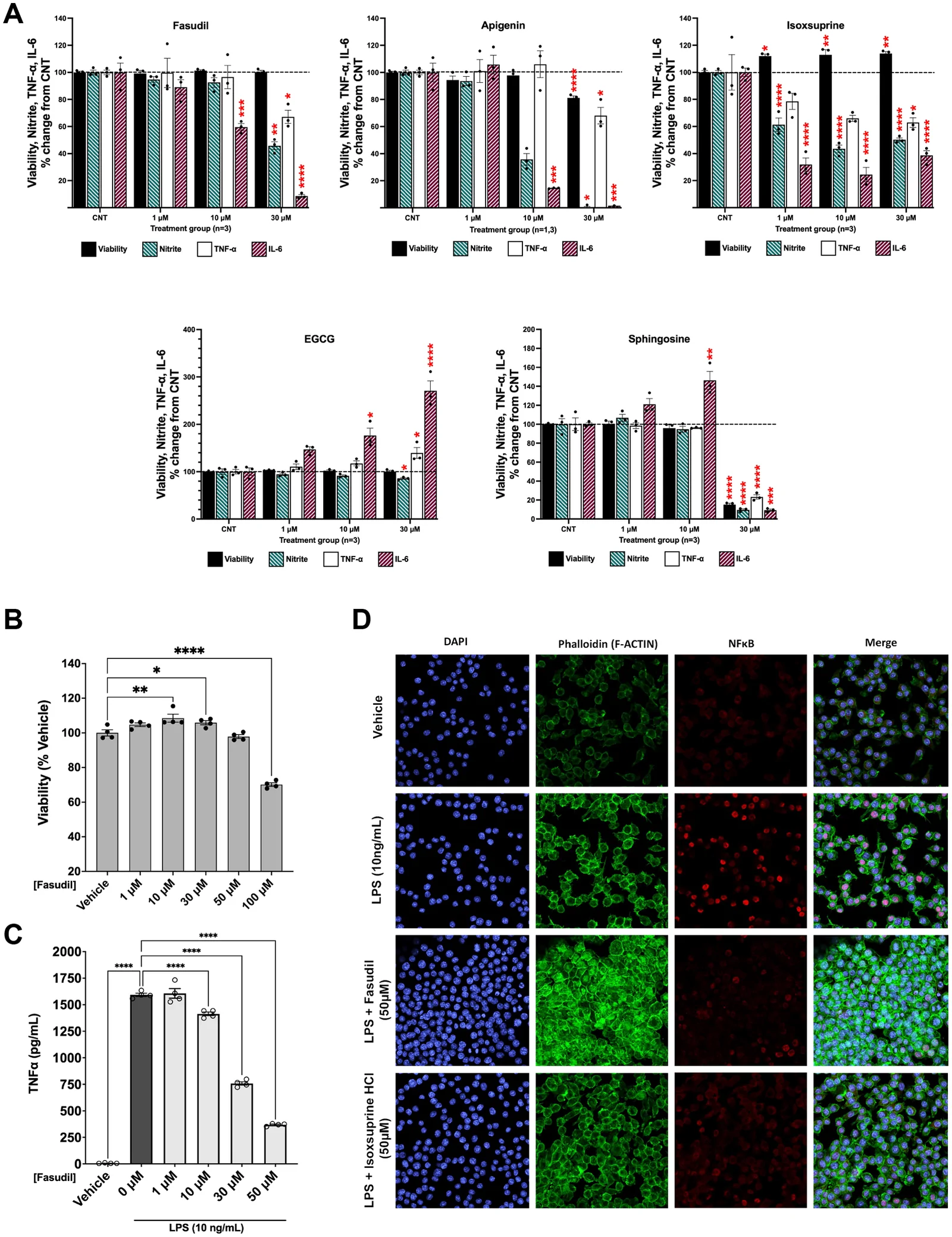
**Effects of fasudil, apigenin, isoxsuprine, EGCG, and sphingosine in LPS-challenged RAW 264.7 cells and IMG cells.** (A) Viability effects and levels of nitrite, TNF-α, and IL-6 in RAW 264.7 cells. Fasudil, apigenin, and isoxsuprine showed strong anti-inflammatory effects, evidenced by reduced media levels of nitrite, TNF-α and IL-6 relative to untreated control (CNT) samples. In contrast, EGCG and sphingosine showed moderate pro-inflammatory effects, evidenced by increased media levels of IL-6 and TNF-α for EGCG. EGCG also showed modest anti-inflammatory activity at 30 µM, evidenced by reduced nitrite levels. Toxicity was observed for sphingosine at 30 µM. (B) Treatment of IMG cells with fasudil (no LPS) showed that fasudil increased cell viability at 10 and 30 µM but was toxic at 100 µM. (C) Fasudil induced dose-dependent reductions in secreted TNF-α in LPS-challenged IMG cells, indicating significant anti-inflammatory effects. (D) LPS-challenged IMG cells showed increased nuclear expression of NF-κB, and fasudil and isoxsuprine at 50 µM both reduced nuclear localization of NF-κB (in red). All data are shown as mean±s.e.m. of observations, *N*=3-4. Statistical significance was determined by ordinary one-way ANOVA with Dunnett's multiple-comparison test or Kruskal–Wallis test with Dunn's multiple-comparison test, with significance indicated as follows: *P<0.05, **P<0.01, ***P<0.001, ****P<0.0001.

In LPS-stimulated RAW 264.7 cells, fasudil, apigenin, and isoxsuprine all showed strong anti-inflammatory effects, evidenced by significant reductions in media levels of nitrite, TNF-α and IL-6 (Fig. 2; Table S3a). Fasudil at 30 µM reduced the levels of nitrite, IL-6 and TNF-α with no cell toxicity, and fasudil at 10 µM also showed reduced IL-6 levels. Apigenin potently reduced nitrite, IL-6 and TNF-α at 30 µM; however, at this concentration, there was evidence of a mild but statistically significant level of cell toxicity. Apigenin at 10 µM reduced levels of nitrite and IL-6 with no cell toxicity. Isoxsuprine strongly reduced all markers of inflammation at all three concentrations with no cellular toxicity (high variability limited the significance of TNF-α reduction at 1 and 30 µM) and also increased viability.

In contrast, EGCG showed both pro- and anti-inflammatory effects, and sphingosine showed modest pro-inflammatory effects in LPS-stimulated RAW 264.7 cells (Fig. 2; Table S3). Both compounds generated elevations in IL-6 in a concentration-dependent manner. These were statistically significant for EGCG at 10 and 30 µM and for sphingosine at 10 µM. Sphingosine at 30 µM proved to be toxic to RAW 264.7 cells (15±1% viability compared to control). Only EGCG at 30 µM was able to reduce nitrite levels with no cellular toxicity.

The anti-inflammatory effects of fasudil and isoxsuprine were subsequently validated in LPS-challenged mouse immortalized microglial (IMG) cells. This immortalized microglial line closely models brain function, is widely used in studies of brain disease (Banerjee et al., 2021), and exhibits potent anti-inflammatory effects from ROCK and pan-kinase inhibitors (Glotfelty et al., 2023). Viability testing of IMG cells treated with fasudil from 1 to 100 µM demonstrated moderate but significant elevations in cell viability up to a concentration of 30 µM (Fig. 2B; Table S3b), which paralleled the increased growth seen following fasudil treatment in NPCs (Fig. 1B). Fasudil at 100 µM demonstrated cellular toxicity; thus, a maximal concentration of 50 µM was used in subsequent LPS studies. Viability testing in LPS-challenged IMG cells confirmed that fasudil remained nontoxic from 1 to 50 µM (*P*=0.2659, ordinary one-way ANOVA).

Fasudil reduced the levels of secreted TNF-α in LPS-challenged IMG cells in a concentration-dependent manner (Fig. 2C; Table S3b). Furthermore, immunofluorescence showed that while LPS stimulation of IMG cells caused elevated nuclear expression of NF-κB, indicating that NF-κB has been released from cytoplasmic sequestration and has entered the nucleus to activate target genes, treatment of IMG cells with fasudil or isoxsuprine prior to LPS stimulation reduced NF-κB nuclear staining (Fig. 2D). These results show that both fasudil and isoxsuprine dampen the NF-κB pathway, a novel finding related to isoxsuprine and consistent with previous findings related to fasudil in other disease models and cell types (Guo et al., 2020; Hou et al., 2012; Liu et al., 2022; Okamoto et al., 2010; Song et al., 2013).

### Fasudil partially corrects dysregulated gene expression and pathways in T21 NPCs

**Fig. 3.**
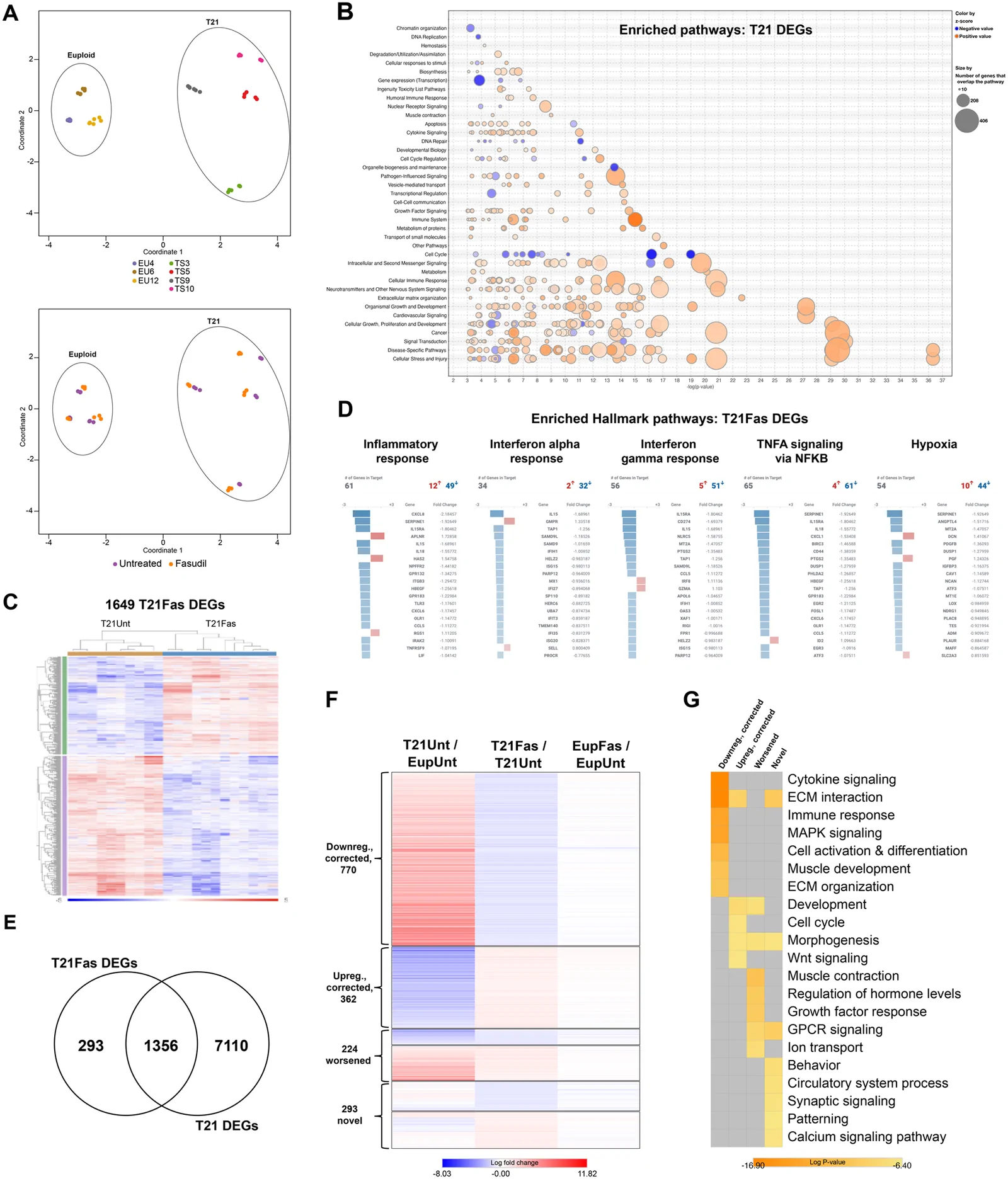
**RNA-seq analysis of fasudil-treated NPCs showed that fasudil partially corrected dysregulated gene expression and immune system pathways in T21 NPCs.** (A) MDS plot of all RNA-seq data for T21 and Eup NPCs treated with fasudil shows strong separation of NPC cell lines by genotype and moderate separation of fasudil-treated samples from untreated samples, with greater separation seen in T21. The top plot is colored by individual NPC lines to show genotype separation, and the bottom plot is colored by fasudil-treated samples (orange) and untreated samples (purple). T21 and Eup lines are named with the prefixes TS and EU, respectively. (B) IPA showed top pathway categories of significantly enriched pathways for T21Unt versus EupUnt NPCs, termed T21 DEGs. Blue=negative *Z*-score/pathway downregulation, and orange=positive *Z*-score/pathway upregulation. The circle size represents the number of DEGs in the pathway. Upregulation of immune system pathways and downregulation of cell cycle pathways are present. (C) Hierarchical clustering of 1649 DEGs identified between T21Fas NPCs and T21Unt NPCs, termed T21Fas DEGs. (D) Heatmaps of the top 20 genes (by |FC|) in significantly enriched MSigDB Hallmark pathways, illustrating downregulation of inflammatory response, interferon alpha and gamma responses, TNF-α signaling by NF-κB, and hypoxia pathways in T21Fas DEGs. (E) Venn diagram showing 1356 DEG overlap between T21Fas DEGs and T21 DEGs. (F) Heatmap of log FC values for the 1649 T21Fas DEGs in T21Unt versus EupUnt (left), T21Fas versus T21Unt (center), and EupFas versus EupUnt (right). Heatmap rows are clustered based upon correction of T21 DEGs by T21Fas DEGs (770 downregulated, corrected, and 362 upregulated, corrected), worsening of T21 DEGs by T21Fas DEGs (224 worsened, middle), and T21Fas DEGs that did not overlap with the T21 DEG set (293 novel, bottom). (G) Pathway analyses of the four sub-clusters of T21Fas DEGs showed that the most significantly enriched pathways are in the downregulated, corrected DEGs and affect immune system functions.

To examine the effects of fasudil treatment on Eup and T21 NPC RNA expression, bulk RNA sequencing (RNA-seq) analysis was performed. Total RNA for RNA-seq was collected at 120 h from Eup and T21 NPC lines (*N*=3 and 4, respectively) that were cultured in the six-well plate format in the presence or absence of fasudil (Fig. 1A). Multidimensional scaling (MDS) of all RNA-seq data showed separation of Eup and T21 NPC lines (Fig. 3A). T21 NPC lines showed more variability than Eup lines by MDS, corresponding with the variability previously observed for these lines by microarray and proteomics analyses (Lee et al., 2025). Genotype comparison of untreated T21 (T21Unt) and untreated Eup (EupUnt) samples identified 8466 DEGs [T21 DEGs, *P*_adj_≤0.05, fold change (FC)≥|1.5|], reflecting the widespread gene expression changes that occur from T21 (Table S4a). Ingenuity pathway analysis (IPA) showed that enriched pathways associated with T21 DEGs included upregulation of cellular stress and injury, cellular immune response, and immune system pathways along with downregulation of cell cycle pathways (Fig. 3B; Table S4b), matching previous analyses of microarray data from these NPC lines (Lee et al., 2025).

Examination of MDS clusters for individual T21 NPC lines showed separation of fasudil-treated and untreated samples (Fig. 3A, bottom). This separation was also apparent in two Eup lines, but to a lesser degree, indicating extensive gene expression changes from fasudil treatment in T21 NPCs. Concordant with this result, differential gene expression analysis between T21Fas and T21Unt samples identified 1649 DEGs (*P*_adj_≤0.05, FC≥|1.5|, Fig. 3C; Table S5a). Twenty-one of these genes were encoded on *Hsa*21 (Table S5b).

The most significantly enriched MSigDB Hallmark pathways in the 1649 DEGs showed downregulation of interferon alpha, interferon gamma, inflammatory response, and TNF-α signaling by NF-κB (Fig. 3D; Table S5c), corresponding with the anti-inflammatory properties observed in RAW 264.7 and IMG cells following fasudil treatment (Fig. 2). The Hallmark hypoxia pathway was also significantly enriched and downregulated (Fig. 3D, Table S5c), which is notable because increased reactive oxygen species have been previously reported in T21 neurons (Briggs et al., 2013; Busciglio and Yankner, 1995). IPA significantly enriched pathways also included numerous cellular immune response and cellular stress and injury pathways, including downregulation of interferon signaling, neuroinflammation, and cGAS-STING pathways (Fig. S3, Table S5d).

To determine if fasudil treatment corrected altered gene expression in T21 cells, the 1649 T21Fas DEGs were compared to the 8466 T21 DEGs, and 1356 overlapping DEGs were identified (Fig. 3E; Table S6a). Interestingly, fasudil partially corrected T21 gene dysregulation in most overlapping DEGs, as 83.5% (1132/1356) of the T21Fas DEGs showed expression changes in the opposite direction from those in T21 DEGs (Fig. 3F; Table S6b), i.e. dysregulated genes in the genotype comparison were attenuated in the T21 fasudil treatment comparison. The 1132 corrected DEGs included 13 *Hsa*21-encoded T21 DEGs (Table S6c). Hierarchical clustering of the FC values for these subsets of genes (Fig. 3F) illustrated that approximately two-thirds of the 1132 corrected genes were downregulated by fasudil (770 genes). Only 224 T21Fas DEGs (16.5%) worsened T21-associated gene expression changes, and the remaining 293 DEGs (17.8%) were novel (Fig. 3F; Table S6a).

Correction by fasudil was also apparent at the pathway level. Comparison of IPA-enriched pathway lists identified 64 T21Unt versus EupUnt pathways (with predicted directionality and an IPA *Z*-score of >|2|) that were rescued by fasudil in T21 NPCs (Table S6d). Most were downregulated by fasudil, including many immune system-related pathways such as interferon alpha/beta signaling, cGAS-STING signaling, interferon gamma signaling, Rho GTPase cycle, and senescence. Rescued pathways that were upregulated by fasudil related to cell cycle and chromosome replication, pathways that are characteristically slowed in T21 cells. Upregulation of Sonic Hedgehog (SHH) signaling was also present. This result is intriguing because SHH pathways, which regulate critical aspects of normal NPC development, are impaired in mouse models of DS, and restoration of SHH expression or downstream signaling can alleviate abnormal neurological phenotypes (Das et al., 2013; Gao et al., 2021; Roper et al., 2006).

Metascape pathway analysis on the four DEG subsets – downregulated, corrected; upregulated, corrected; worsened; and novel (Fig. 3F) – showed that the most significantly enriched pathways were in the downregulated, corrected DEG set (Fig. 3G). Two of the top downregulated pathway classes were cytokine signaling and immune response, consistent with fasudil correcting the elevated immune system pathways in T21 NPCs (Fig. 3G). The upregulated, corrected DEGs were enriched for development and cell cycle pathways, corresponding to the increased growth seen with fasudil treatment (Fig. 3G). The worsened DEG pathways had less significant average *P-*values than the downregulated, corrected pathways and included pathways related to muscle contraction, regulation of hormone levels, and growth factor response. Novel DEGs were enriched for a variety of pathways, including G protein-coupled receptor signaling, behavior, and circulatory system processes. Taken together, these transcriptomic analyses indicate that fasudil causes widespread and primarily beneficial changes in T21 gene expression, with the strongest effects seen in reduction of elevated immune system pathways.

The fasudil-induced gene expression changes in Eup NPCs differed from those in T21 NPCs. FC values for fasudil-treated Eup (EupFas) versus EupUnt NPCs using the 1649 T21Fas DEG set indicated no or minimal expression changes in these genes in Eup NPCs (Fig. 3F, right). Differential gene expression analysis between EupFas and EupUnt NPCs identified only 172 DEGs, which was approximately tenfold fewer than those in T21 lines (Table S7a). Unlike T21 NPCs, pathway enrichment analyses for Eup NPCs did not identify downregulation of inflammatory pathways from fasudil treatment. Upregulation of *WNT1*, *WNT10B*, *WNT2B*, *WNT3A*, and *WNT4* expression was present in EupFas NPCs, leading to enrichment of multiple WNT signaling pathways regulating growth, proliferation, and development (Table S7b).

**Fig. 4.**
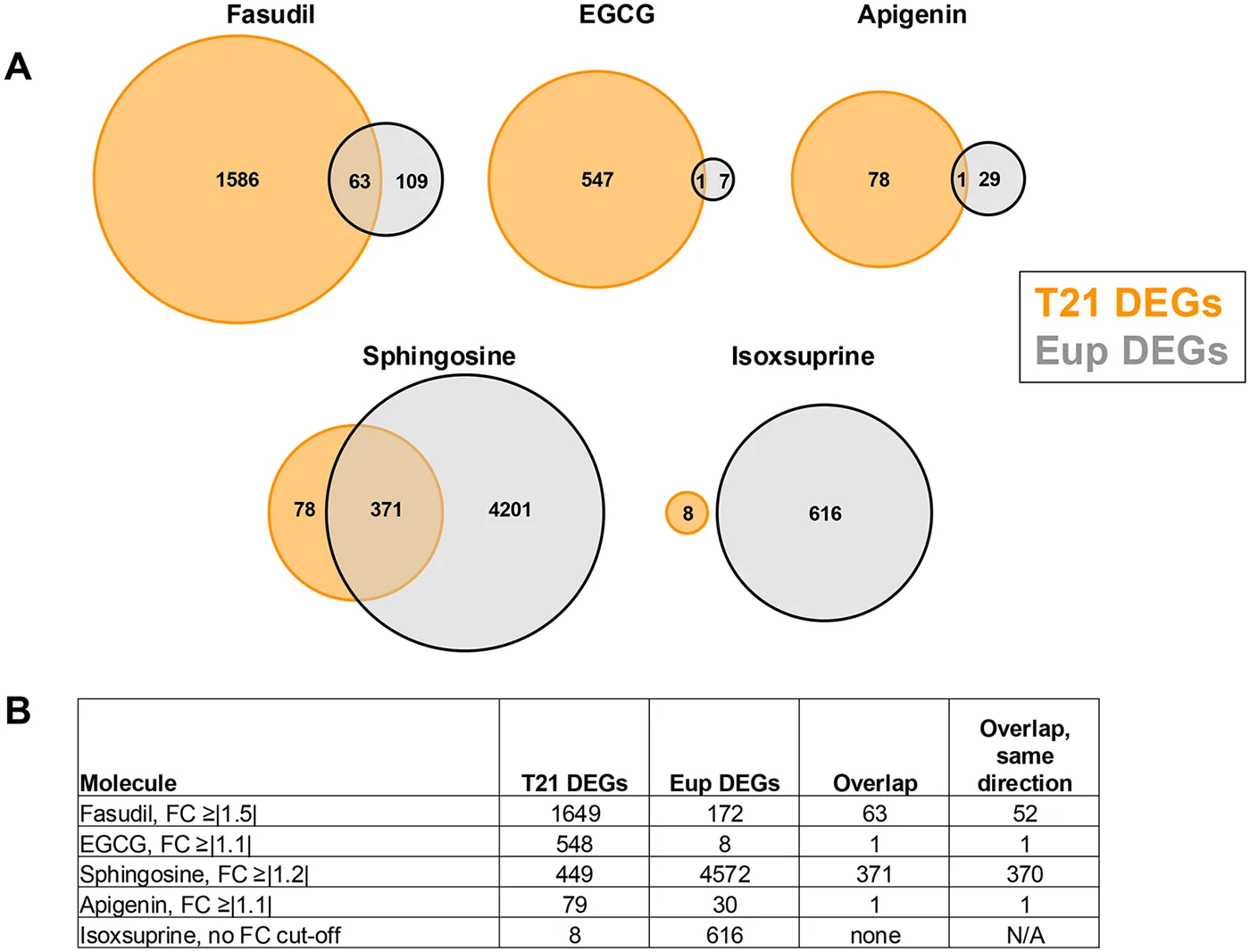
**Eup and T21 NPCs showed different transcriptomic responses to candidate pharmacotherapies.** (A) Venn diagrams illustrating that T21 NPCs (orange) showed greater numbers of DEGs than Eup NPCs (gray) in response to fasudil, EGCG, and apigenin. In contrast, Eup NPCs showed more DEGs in response to sphingosine and isoxsuprine. (B) Table of DEGs for T21, Eup NPCs, DEGs that overlap between T21 and Eup, and overlapping T21 and Eup DEGs that changed in the same direction in response to each candidate molecule. All DEGs used a cutoff of *P*_adj_≤0.05, and FC cutoff values are shown for each molecule's DEG set. For isoxsuprine, most FC values were <|1.1|, so no FC cutoff was used.

Comparison of the T21Fas and EupFas DEG gene sets (1649 and 172 DEGs, respectively) identified 63 shared DEGs, 52 of which were changed in the same direction by fasudil (Fig. 4A; Table S7c). This shared list included upregulation of genes encoding the transcription factors LMXA and DMBX1, the enzyme DCT, and the signaling molecules BMP4 and WNT4, all of which regulate NPC proliferation and forebrain development*.* Most of the DEGs that were changed in the same direction (40/52, 77%) showed greater FC values in T21 (Table S7c), which may reflect the stronger growth effects observed in T21 (Fig. 1C).

### Effects of EGCG, apigenin, isoxsuprine and sphingosine on T21 NPCs

Bulk RNA-seq analyses were also performed on total RNA from Eup and T21 NPCs that were treated with EGCG, apigenin, isoxsuprine, or sphingosine. Differential gene expression analysis was first performed between treated and T21Unt NPCs for each candidate pharmacotherapy.

In contrast to fasudil's effects, EGCG showed modest effects on gene expression in T21 NPCs, indicated by only one DEG at *P*_adj_≤0.05, FC≥|1.5|, and 30 DEGs at *P*_adj_≤0.05, FC≥|1.2|. Therefore, a statistical cutoff of *P*_adj_≤0.05, FC≥|1.1|, was used, giving 548 T21EGCG DEGs (Table S8a). Enriched pathway analysis showed upregulation of multiple pathways related to extracellular matrix organization and cell surface interaction (Table S8b). In addition, most EGCG-induced gene expression changes did not correct T21 dysregulation: 374 T21EGCG DEGs overlapped with the 8175 T21 DEGs (untreated cells in the EGCG experiment, Table S8c), and 84.5% of these showed FC values in the same direction (316/374) (Table S8d). Enriched pathway analysis of the overlapping 374 DEG set (Table S8e) showed upregulation of multiple pathways governing cancer/cell growth, extracellular matrix organization, and cellular stress and injury. Several pathways related to the cellular immune response/immune system were also upregulated, which may correspond to the modest pro-inflammatory effects of EGCG that were seen in LPS-stimulated RAW 264.7 cells. Overall, these results indicate that EGCG caused modest changes in T21 NPC gene expression, and these changes primarily worsened T21-associated gene dysregulation.

Sphingosine treatment of T21 NPCs also showed more modest changes in gene expression in comparison to fasudil, with fewer DEGs and smaller FCs. In sphingosine-treated T21 (T21Sph) NPCs relative to T21Unt NPCs, only six DEGs were present, using a cutoff of *P*_adj_≤0.05, FC≥|1.5|; thus, a limit of *P*_adj_≤0.05, FC≥|1.2|, was applied, which gave 449 DEGs (Table S9a). This gene list included upregulation of multiple genes encoding collagens and matrix metalloproteinases. IPA and Metascape pathway analyses of the 449 DEG set identified upregulation of multiple pathways related to extracellular matrix organization as well as enrichment of pathways regulating synaptic transmission, development, and behavior (Table S9b,c). Approximately half of the T21Sph versus T21Unt DEGs (244/449 DEGs) overlapped with the 5127 T21DEGs (untreated cells in the sphingosine experiment, Table S9d), and 85% of the sphingosine-induced gene expression changes showed FC values in the same direction as the T21DEGs (207/244), thus worsening T21-associated gene expression changes (Table S9e). The shared DEG list included upregulation of *TGFB1*, of note because increased TGFB1 expression occurs in T21 iPSC-derived NPCs and has been suggested to contribute to the altered balance between neurogenesis and gliogenesis in T21 NPCs (Huang et al., 2025). Enriched pathway analysis of the overlapping 244 DEG set (Table S9f) identified upregulation of pathways related to extracellular matrix organization, pathogen-induced cytokine storm signaling, and HIF-1 signaling. Overall, these results show no clear improvements in T21 gene dysregulation and indicate that sphingosine may exacerbate T21-associated gene dysregulation in NPCs.

Apigenin and isoxsuprine showed minimal effects on gene expression in T21 NPCs. For apigenin, there were no DEGs (*P*_adj_≤0.05) with FC values of ≥|1.2|, and only 79 DEGs showed FC values of ≥|1.1| (Table S10a). Only eight DEGs were present for isoxsuprine, and these DEGs had extremely low FCs (<|1.1|) (Table S10b). The small numbers of genes in the apigenin and isoxsuprine T21 DEG lists prevented enriched pathway analyses.

### Eup and T21 NPCs show different responses to candidate pharmacotherapies

As mentioned above, T21 NPCs showed more transcriptional changes than Eup NPCs in response to fasudil. Interestingly, differential gene expression analysis between treated Eup and EupUnt NPCs for each candidate pharmacotherapy (Table S11a-d) revealed that the magnitude of transcriptomic changes for T21 and Eup NPCs also differed for the other four candidate therapeutic molecules (Fig. 4). T21 NPCs showed more DEGs than Eup in response to fasudil, EGCG, and apigenin, and Eup NPCs showed more DEGs than T21 in response to sphingosine and isoxsuprine (Fig. 4A,B). Furthermore, there was little to no overlap of T21 and Eup DEGs in EGCG, apigenin, or isoxsuprine, indicating distinct transcriptional effects by genotype. Although there was a greater overlap of DEGs in fasudil and sphingosine and the majority of DEGs changed in the same direction for both T21 and Eup, there were striking differences in the number of DEGs between T21 and Eup for these two molecules, with approximately tenfold more DEGs in T21 NPCs for fasudil and approximately tenfold more DEGs in Eup NPCs for sphingosine. Of note, pathway analyses of the sphingosine Eup DEGs identified significant upregulation of a variety of immune response pathways, including interferon alpha and interferon gamma responses, inflammatory response, and TNF-α signaling by NF-κB (Table S11e,f), supporting the pro-inflammatory effects of sphingosine in Eup cells seen in RAW 264.7 cells. Taken together, these results suggest that the transcriptomic responses of T21 NPCs to pharmacotherapies may differ from what is expected based upon Eup NPC responses.

## DISCUSSION

The potential of therapeutic treatments to alleviate deleterious effects of T21 on early human neurodevelopment is unexplored, and studies using human model systems are sorely needed to address this research gap. This study used *in vitro* cultures of Eup and T21 NPCs to evaluate the ability of candidate pharmacotherapies to alleviate T21-associated cellular phenotypes that may be relevant to atypical neurodevelopment in DS. The ROCK inhibitor fasudil consistently increased proliferation in T21 NPCs, suggesting that fasudil could improve the reduced proliferation that is seen in T21 neurodevelopment. Interestingly, fasudil's effects were more pronounced on slow-growing T21 NPC lines; while these results require future validation, it may reflect fasudil's ability to directly target pathways governing proliferation that are dysregulated by T21. Fasudil also demonstrated anti-inflammatory effects, evidenced by reduced RNA expression of inflammatory pathway genes in T21 NPCs and reduced inflammatory markers in LPS-challenged microglia model systems. Previous studies have shown that numerous phenotypes in mouse models of DS and individuals with T21 are associated with upregulated interferon and inflammatory pathways, indicating the relevance of candidate therapeutics with anti-inflammatory properties (Chi et al., 2023; Galbraith et al., 2023; Rachubinski et al., 2024; Sullivan et al., 2017, 2016; Tuttle et al., 2020; Waugh et al., 2023). Furthermore, fasudil treatment upregulated genes involved in SHH signaling, which is essential for proper neurogenesis throughout the developing brain (Belgacem et al., 2016). Previous studies in the Ts65Dn mouse model of DS revealed deficits in SHH signaling (Gao et al., 2021). Furthermore, restoring the SHH pathway in the early prenatal period improved cellular proliferation in the cerebellum and corrected behavioral deficits in learning/memory and hyperactivity (Das et al., 2013; Gao et al., 2021; Roper et al., 2006). Fasudil's amelioration of SHH pathway gene dysregulation suggests that fasudil administration during early neuronal development might also improve these cell growth and behavioral phenotypes. Overall, the results of this study demonstrate fasudil's potential to simultaneously target multiple T21-associated abnormalities in a beneficial manner.

Our *in vitro* results using human T21 NPCs complement those of an *in vivo* fasudil study using the Ts65Dn mouse model of DS (López-Hidalgo et al., 2024). Chronic treatment of Ts65Dn mice with fasudil from postnatal day (P) 0 to P28 improved cell proliferation and neurogenesis of the subgranular zone of the hippocampus, corrected abnormal dendritic morphology of trisomic hippocampal neurons, and reduced inhibitory synapses, which are more numerous in both Ts65Dn and T21 (López-Hidalgo et al., 2024). Thus, different preclinical model systems and outcome measures both point to the potential therapeutic effects of fasudil on neurological development.

Fasudil inhibits both ROCK1 and ROCK2, affecting multiple ROCK downstream targets. Therefore, fasudil and other ROCK inhibitors have the potential to alleviate numerous pathologies, including neurodegeneration, cancer, glaucoma, and kidney failure (Cheng et al., 2025; Feng et al., 2016). Fasudil has been used clinically in Japan since 1995 to prevent cerebral vasospasm after subarachnoid hemorrhage (Roskoski, 2023; Shibuya et al., 1992; Zhao et al., 2006). The beneficial effects of fasudil in preclinical models of neurodegenerative disorders (Koch et al., 2018; Song et al., 2013; Takata et al., 2013; Yang et al., 2020), which may relate to its ability to block immune system/microglial activation (Roser et al., 2017; Wang et al., 2022), have led to fasudil's employment in multiple clinical trials related to neurodegeneration (ClinicalTrials.gov) (Koch et al., 2024; Wolff et al., 2024a,b). Of note, fasudil shows inhibitory effects on multiple protein kinase pathways in addition to ROCK1 and ROCK2 (https://www.guidetopharmacology.org/GRAC/LigandScreenDisplayForward?ligandId=5181&screenId=3). This concern is being addressed by the development of more selective analogs such as ripasudil (Cheng et al., 2025; Feng et al., 2016; Koch et al., 2018; Ye et al., 2024).

ROCKs perform essential functions during earlier embryonic developmental stages and organogenesis (Shi and Wei, 2022), and thus, application of fasudil at these early time points could be detrimental. However, animal studies suggest the feasibility of prenatal administration of fasudil during later gestational stages, which would align with human fetal stages that occur after diagnosis of T21. In rats, fetal exposure to fasudil reduced preeclampsia (Gu et al., 2017) and reduced intrauterine growth restriction, although this was also associated with greater postnatal body weight, increased food intake, and metabolic changes (Butruille et al., 2012). Fasudil also reduced the myogenic (vasoconstriction) response in acute ductus arteriosus compression in late gestation fetal sheep (Tourneux et al., 2008).

Anti-inflammatory properties have been previously reported for fasudil (Guo et al., 2020; Hou et al., 2012; Liu et al., 2022; Okamoto et al., 2010; Song et al., 2013), EGCG (Capasso et al., 2025), apigenin (Guedj et al., 2020), and downstream metabolic products of sphingosine (Gomez-Larrauri et al., 2025). To the best of our knowledge, the anti-inflammatory effects of isoxsuprine described in this study are novel. While anti-inflammatory effects of fasudil were identified in T21 NPCs as well as LPS-challenged microglial systems, isoxsuprine showed only inflammatory effects in the microglial studies. The different anti-inflammatory properties of candidate pharmacotherapies may reflect cell-specific or developmental-stage-specific effects of each molecule that will need to be elucidated further. Microglia may play important roles in T21 CNS phenotypes (Flores-Aguilar et al., 2020; Jin et al., 2022; Martini et al., 2020; Pinto et al., 2020; Tiberi et al., 2025), so additional studies on trisomic microglia would be warranted. Interestingly, recent studies showed transcriptomic changes indicating microglial activation in the prenatal T21 cortex (Risgaard et al., 2026; Vuong et al., 2026), suggesting that microglia may be a highly relevant target alongside neurons for candidate prenatal therapeutics in DS.

Although EGCG and sphingosine showed fewer T21 DEGs in comparison to fasudil, most gene changes for EGCG and sphingosine were predicted to worsen T21-associated gene dysregulation, suggesting that these molecules would not benefit neurodevelopment and could potentially cause harm. This is of interest because some parents administer dietary supplements such as EGCG/green tea extract to their children with DS, often without informing medical caregivers (Lewanda et al., 2018). EGCG studies have shown mixed results in individuals with T21 and mouse models of DS. While several studies report beneficial effects on skeletal/craniofacial, cognitive, behavioral, and cardiac phenotypes (Blazek et al., 2015; Catuara-Solarz et al., 2015; de la Torre et al., 2016, 2014; McElyea et al., 2016; Souchet et al., 2019; Stagni et al., 2016; Starbuck et al., 2021; Tielemans et al., 2025), other studies showed no effects (Cieuta-Walti et al., 2022), deleterious effects (Goodlett et al., 2020; Tielemans et al., 2025), or variable effects related to dosage, timing of administration, or type of commercially available supplement (Abeysekera et al., 2016; Jamal et al., 2022; Stringer et al., 2017). Controlled preclinical and clinical studies are needed to understand the complex effects of these molecules and thus allow clinicians to advise parents in an informed manner.

The transcriptomic-based approach used in this study did not identify new molecules with beneficial effects on cell growth or inflammation in NPCs. While this may reflect limitations of the testing used in our NPC model system, it may also relate to distinct transcriptional responses of Eup and T21 cells to the same molecule. Eup and T21 NPCs showed strikingly different gene expression responses for all five pharmacotherapies examined by RNA-seq in this study. This suggests that T21 cells may respond to candidate therapeutics in ways that cannot be predicted from Eup cells, and thus, Eup-cell-derived gene signatures may not accurately predict the pharmacological effects on transcription in T21 cells. Future queries of LINCS with targeted subsets of T21 DEGs – for example, gene signatures that include only inflammatory or oxidative stress pathways – may be more informative than queries using the full gene expression signature. Newer deep learning methods for predicting treatment responses that could be trained on these new data from T21 NPCs might also improve cell-specific connectivity results. In addition, the negative effects on cell growth for eight candidate molecules (Table S1) could have been caused by targeting T21 gene dysregulation that promotes survival in the context of aneuploidy. If true, these essential T21 pathways will need to be defined in future studies so that they can remain unchanged during the application of pharmacological treatments.

In summary, we demonstrated that Eup and T21 NPCs showed distinct transcriptomic responses to five candidate pharmacotherapies. We also determined that the ROCK inhibitor fasudil improved growth in T21 NPCs, normalized expression of inflammatory genes in T21 NPCs, and reduced inflammatory markers in LPS-stimulated microglial model systems. Our results, combined with those from a prior *in vivo* study (López-Hidalgo et al., 2024), suggest that fasudil merits serious consideration for further study as a potential prenatal pharmacotherapy.

## MATERIALS AND METHODS

### Selection of candidate pharmacotherapies using LINCS

To identify novel therapeutic candidate molecules via a transcriptomics approach, connectivity mapping was performed on a connectivity database created from a subset of the 2017 LINCS GEO releases, reflecting 80 cell types and 1330 named molecules, previously described as the larger ‘sparse’ matrix (Sapashnik et al., 2023). This dataset was then completed by imputing missing data with the neighborhood collaborative filtering method, as described in Section 3.5 of Sapashnik et al. (2023), to provide data for any cell/molecule combinations not experimentally evaluated in the LINCS data. The query signature dataset contained 76 upregulated and 58 downregulated genes (Table S1e), which corresponded to all genes within the T21 NPC DEG list [false discovery rate (FDR)-adjusted *P*=0.1, FC≥|1.2|] previously published by our lab (Lee et al., 2025) that are also represented on the L1000 platform.

The 165 molecules with LINCS scores <−0.2 in NPCs were screened for toxicity and teratogenicity by searching PubMed for published articles on each molecule using the molecule name and toxic or teratogenic as search terms. Molecules with reported teratogenicity or contraindications in pregnancy were eliminated from consideration for further testing, and only molecules with no or limited toxic effects and limited or mild adverse effects were used for testing. A subset of 17 molecules from this list was chosen for further examination using NPC cultures.

### Cell culture

Nine Eup and eight T21 iPSC lines previously generated in our laboratory (Lee et al., 2025) were used for these experiments (Table S2). iPSC lines were stably transduced with Nuclight Red Lenti, EF1a, puro (4625, Sartorius, Gottingen, Germany) per the manufacturer's instructions, which generated a nuclear-restricted fluorescent signal that enabled live-cell imaging. Stable selection for the Nuclight Red vector was performed using 1 µg/ml of puromycin, and subsequent maintenance was performed using 0.5 µg/ml of puromycin. iPSCs were cultured on Corning Matrigel-coated plates (354277, Fisher Scientific, Waltham, MA, USA) in Gibco StemFlex media (A3349401, Thermo Fisher Scientific), and colonies were passaged as aggregates at 60-70% confluency using ReLeSR (100-0483, STEMCELL Technologies, Vancouver, BC, Canada).

Neural induction of stably transduced iPSCs to generate NPCs was performed as previously described (Lee et al., 2025). Briefly, Nuclight Red-transduced Eup and T21 iPSC lines were dissociated to single cells at passage 10 or greater following transduction using Accutase (07920, STEMCELL Technologies) and plated at 1×10^5^ cells/cm^2^ on Matrigel-coated plates using the STEMdiff SMADi Neural Induction Kit (08581, STEMCELL Technologies). All subsequent passages used Accutase. After being cultured for three passages in STEMdiff Neural Induction Media, cells were switched to STEMdiff Neural Progenitor Media (05833, STEMCELL Technologies), and lines were subsequently maintained on Matrigel in this medium. After three passages, NPCs were cryopreserved in CryoStor CS10 (100-1061, STEMCELL Technologies) at each passage up to passage 10. All NPC assays were performed between passages 3 and 10, with most assays performed between passages 5 and 7.

At each passage during neural induction and NPC maintenance, medium was supplemented with a CEPT cocktail (Chen et al., 2021) at the following final concentrations: 50 nM chroman 1 (7163, Bio-Techne, Minneapolis, MN), 5 µM emricasan (S7775, Selleck Chemicals), 1:1000 dilution of polyamines (P8483, MilliporeSigma), and 0.7 µM trans-ISRIB (5284, Bio-Techne). Chroman 1 is a ROCK inhibitor, emricasan is a pan-caspase inhibitor, polyamines support cell growth and cell attachment, and trans-ISRIB selectively inhibits the integrated stress response. Media containing CEPT was removed within 24 h of passaging and replaced with unsupplemented media.

Maintenance of the expected T21 karyotype in trisomic lines, along with the absence of additional chromosomal abnormalities, was confirmed in NPCs by KaryoStat analyses (Thermo Fisher Scientific). NPC cultures were confirmed to be free of mycoplasma contamination by PCR testing of media using a mycoplasma PCR detection kit (G238, Applied Biological Materials, Richmond, BC, Canada).

### Analysis of the effects of CEPT and Y-27632 on growth

Three Eup lines (EU6, EU7, and EU12) and three T21 iPSC lines (TS3, TS4, and TS8) were cultured in six-well plates and subjected to neural induction, as described above. One cell line was plated per plate at 1×10^5^ cells/cm^2^ at each passage. Three wells were passaged with media containing CEPT and the other three with media containing 10 µM Y-27632 (*n*=3 for each condition). Cells were cultured for three passages, and growth from passage 0 through passage 3 was monitored by live-cell imaging of fluorescent nuclei at 594 nM every 24 h. Imaging was performed using a Cytation 5 Cell Imaging Multimode Reader (Agilent Technologies, Santa Clara, CA, USA), and cell counts were measured using Gen5 version 3.12 imaging software (Agilent Technologies), with object sum area of fluorescent nuclei values used to measure the cell number. Ratios of CEPT-treated cell counts to Y-27632 cell counts were calculated for each cell line at 1, 2, and 3 days after each passage.

### Quantitation of NPC growth in the presence of candidate pharmacotherapies

Eup and T21 NPCs were plated with NPC media containing CEPT at an initial density of 5000 cells/well (1.0E+05 cells/ml) in 384-well black optical-bottom plates (164586, Thermo Fisher Scientific) or 75,000 cells/well (3.75E+04 cells/ml) in standard six-well tissue culture plates. The six-well plate studies were performed after the 384-well plate studies to examine molecule effects in a larger-well format in which more consistent cell growth could be achieved over longer time periods and sufficient RNA could be collected for RNA-seq.

NPCs were cultured for 120 h in the presence or absence of candidate pharmacotherapeutic molecules. At 12 h, medium was changed to remove CEPT and was replaced with either NPC media containing candidate molecules (treated wells) or unsupplemented NPC media (untreated wells). This media change was repeated 48 h later (at 60 h). Two or three concentrations of each molecule were used, based on previous *in vitro* dose-range-finding studies of each molecule (Table S1). Each molecule was tested in two independent plates/experiments in four replicate wells per line/condition for the 384-well plate format and in six replicate wells per line/condition for the six-well plate format. Quantitative imaging was performed using a Cytation 5 Cell Imaging Multimode Reader (Agilent Technologies) and BioSpa 8 automated incubator (Agilent Technologies) system along with Gen5 version 3.12 software (Agilent Technologies) to calculate the object sum area of fluorescent nuclei every 12 h for 384-well plate cultures and every 24 h for six-well plate cultures. Subtractive normalization was performed on treated wells using the untreated well object sum area values in the same experiment/plate for each NPC line.

DT was calculated by the equation DT=ln(2)/k, in which the growth rate (k) was calculated by the polyfit function in Python using the least squares method and the general equation for cell growth [*N*(*t*)=(*N*_0_)×kt, where *N* is the cell number/object sum area at a specified time point, *N*_0_ is the initial cell number/object sum area, and *t* is the time]. Outliers were removed by (1) not meeting a minimum threshold of continuous growth for ≥48 h, (2) by using the interquartile range (IQR) outlier formula for DT for each set of four or six replicates (IQR=q3–q1; lower limit=q1−1.5×IQR, upper limit=q3+1.5×IQR), or (3) not meeting a minimum data point count of three for each set of four or six replicates (for 384-well or six-well experiments, respectively) following the previous two outlier removal steps. DT calculations and outlier analyses were done using a Jupyter notebook written in Python. Within-plate comparisons of DTs for untreated and treated wells of the same cell line were tested by the Mann–Whitney *U* test in Python and in GraphPad Prism, with a significance threshold of *P*<0.05. Two-way ANOVAs of growth curves were performed using GraphPad Prism.

### RAW 264.7 cell culture and inflammation assays

The mouse monocyte/macrophage-like cell line RAW 264.7 was purchased from ATCC (TIB-71) and cultured following ATCC recommendations in DMEM+10% FBS (10569-010 and 16140-071, Thermo Fisher Scientific). Compounds were assessed in two studies: (1) treatment with no LPS to evaluate compound tolerability/compound-induced cytotoxicity (1, 10 and 100 µM) and (2) treatment at 1, 10 and 30 µM followed by exposure to LPS (10 ng/ml) to mitigate LPS-induced inflammation. Cells were plated in 24-well plates at a density of either 187,000 (study 1) or 240,000 cells per well (study 2). The next day, cells were pretreated with fasudil, apigenin, EGCG, isoxsuprine HCl, and sphingosine using the vehicle DMSO (Sigma, cat. #D2650), and control cells were pretreated with the same dilution of DMSO. 1 h following the addition of compound/vehicle, the cells were challenged with 10 ng/ml LPS (L4005, Sigma-Aldrich, St. Louis, MO, USA; *Escherichia coli* O55:B5), a dose that induced a substantial and sub-maximal inflammatory response in prior pilot studies (Tweedie et al., 2011, 2009). After 20-24 h, the medium was collected and used for downstream assays. Cell viability was determined using the CytoTox-ONE Homogeneous Integrity Assay (G7890, Promega), culture medium nitrite levels were assessed using the Nitrate/Nitrite Fluorometric Assay Kit (KA1344, Abnova), and culture medium levels of the cytokines TNF-α and IL-6 were determined using ELISAs: ELISA MAX™ Deluxe Set Mouse TNF-α (430915, BioLegend Inc.) and ELISA MAX™ Deluxe Set Mouse IL-6 (431315, BioLegend Inc.). All assays were performed as directed by the manufacturers. Data were normalized to the control LPS+DMSO group, i.e. inflammation without drug treatment, and then expressed as percent change from the control cells, with control data defined as 100%. Data are presented as mean±s.e.m. of *N* observations (*N*=3, except for apigenin IL-6, 30 µM, where *N*=1). Data were processed by GraphPad Prism, version 10.0.3. Values were assessed for outliers (ROUT) and normal distributions (Shapiro–Wilk test). Treatment and control values were compared using ordinary one-way ANOVA followed by Dunnett's multiple-comparison test or Kruskal–Wallis test ANOVA followed by Dunn's multiple-comparison test.

### IMG cell culture and inflammation assays and immunocytochemistry (ICC)

The immortalized adherent mouse microglial ‘IMG’ cell line (SCC-134, Sigma-Aldrich; RRID:CVCL_HC49) was cultured in high-glucose DMEM (D6546, Sigma) with 10% heat-inactivated FBS (10082147, Gibco), 1× L-glutamine (TMS-002-C, Sigma) and 100 U/ml penicillin/streptomycin (15140148, Gibco). IMG cells were cultured according to the product datasheet, with cell passaging performed with Accutase (A6964, Sigma). Cells were used for a maximum of ten passages.

IMG cells for cytokine assays were grown in 24-well (40,000 cells/well) tissue culture-treated plates in 500 µl of media. Cells used for ICC were plated in 96-well sterile glass-bottom plates (P69-1.5H-N, Cellvis) at a density of 30,000 cells/well and were grown to ∼80% confluency (1-2 days). Fresh medium was added on day 1 after plating and changed every 2 days, as needed. To assess possible toxicity induced by fasudil HCl (S1573, Selleck Chemicals), initially, IMG cells were cultured in the presence of fasudil alone (no LPS) in 24-well plates in 500 µl of basic growth media. The following day, cells were treated with fasudil prepared in the same cell culture media (vehicle) at 1, 10, 30, 50 or 100 µM. After 24 h, the medium was collected and replaced with 500 µl of fresh media to allow assessment of cell viability. Fasudil was toxic to IMG cells at 100 µM (compared to vehicle control); thus, 50 µM was the highest concentration used in LPS-induced inflammation-related studies. IMG cell viability was quantified using the CellTiter 96^®^ AQueous One Solution Cell Proliferation Assay kit (MTS) (G3580, Promega, Madison, WI, USA) according to the manufacturer's protocol. IMG cells were pretreated with fasudil at 1, 10, 30, or 50 µM and then, 1 h later, challenged with LPS at 10 ng/ml in the continued presence of fasudil. Following a 24-h incubation, conditioned medium was collected and replaced with 500 µl of fresh basic growth media to allow the assessment of cell viability. Conditioned medium was later analyzed for TNF-α levels by ELISA using the Mouse TNF-α ELISA MAX™ Deluxe Set (430904, BioLegend Inc.), according to manufacturer recommendations. Data are presented as mean±s.e.m. of *N* observations (*N*=3-4). Data were processed by GraphPad Prism, and statistical analysis involved ordinary one-way ANOVA with Dunnett's comparison (versus vehicle or 0 µM fasudil).

For ICC, experimental conditions were replicated in triplicate. Following 1 h of pretreatment with fasudil (50 µM), isoxsuprine HCl (50 µM) (S5669, Selleck Chemicals), or vehicle (media), 10×LPS (final concentration 10 ng/ml) was added for 15 min. Cells were then washed twice with ice-cold PBS and fixed with cold 4% PFA (AAJ19943K2, Fisher Scientific) for 10 min. PFA was removed, and cells were washed three times with cold PBS and stored at 4°C in PBS prior to staining. Cells were permeabilized and blocked in microglial staining buffer (MSB) [3% BSA and 0.1% saponin (47036, Sigma) in PBS] for 1 h at room temperature (RT), followed by incubation with the primary antibody anti-NF-κB (6956S, Cell Signaling, dilution 1:500, raised in mouse) in MSB for 1 h at RT. After primary antibody incubation, cells were washed three times with 0.1% saponin in PBS (wash buffer) for 5 min/wash. The corresponding highly cross-adsorbed Alexa Fluor™ secondary antibody goat anti-mouse 555 IgG (A-21422, Invitrogen, RRID:AB_2535844) was diluted (1:500) in MSB and applied to cells for 1 h at RT. Primary phalloidin staining (A12379, Invitrogen, 488 fluorophore, dilution 1:500) was performed with secondary antibody incubation. Following secondary antibody staining, cells were stained with DAPI (D3571, Invitrogen, dilution 1:1000) for 10 min to visualize cell nuclei (at 405 nm wavelength of excitation). Cells were washed 3×5 min in a wash buffer with a fourth wash in PBS alone. Cells were stored in 100 µl of PBS for imaging. Cells were imaged at 20× using a Nikon Eclipse Ti2 confocal microscope. A *Z*-stack was collected through each cell culture and collapsed into a maximum-intensity projection for the field of view (FOV) (1024 µm×1024 µm). A representative single FOV is shown for each experimental group. Imaging parameters were calibrated using secondary-only controls to set laser power and gain.

### RNA isolation and RNA-seq

Eup and T21 NPCs that had been cultured in the six-well plate format were used for RNA collection. On the final day of culture, medium was removed, and 450 µl of TRI Reagent from Direct-zol Miniprep kits (R2053-A, Zymo Research, Irvine, CA, USA) was added to each well. A cell scraper was used to make a homogeneous lysate, and then samples were pipetted into Eppendorf tubes and stored at −80°C in TRI Reagent until RNA collection. Total RNA was subsequently collected from three untreated and three treated wells for each NPC line using Direct-zol Miniprep kits, following the manufacturer's instructions. RNA concentration and purity were determined using a Nanodrop 8000 (Thermo Fisher Scientific), and RNA integrity (RIN) scores were generated using a Bioanalyzer (Agilent). Most RNA samples had RIN scores of ≥9, and only samples with RIN scores of ≥8.4 were used for sequencing.

1000 ng of total RNA for each sample was used for RNA-seq. Strand-specific, poly-A selected RNA-seq libraries were generated using NEBNext Ultra™ II Directional RNA Library Prep Kits for Illumina and NEBNext Poly(A) mRNA Magnetic Isolation Modules (New England Biolabs). Each library was tagged with unique dual indexes. Sequencing of the pooled libraries was performed at the NIH Intramural Sequencing Center on a NovaSeq X Plus Sequencing System (Illumina) on 25B flow cells. At least 40 million 150-base read pairs were generated for each individual library.

### RNA-seq analyses

Data were analyzed by ROSALIND (San Diego, CA, USA, https://rosalind.bio/). Reads were trimmed using cutadapt1. Quality scores were assessed using FastQC2. Reads were aligned to GRCh38 using STAR3. Individual sample reads were quantified with HTSeq4 and normalized via relative log expression using DESeq2 R library5. Sample MDS plots were generated as part of the QC step using RSeQC6. DESeq2 was used to calculate FCs and *P*-values and to perform optional covariate correction. Clustering of genes for the final heatmap of DEGs was done using the partitioning around medoids method using the fpc R library7. Samples that did not pass quality control were removed prior to differential gene expression analysis. Four Eup and three to four T21 NPC lines were used for each RNA-seq analysis.

The cell line was used for covariate correction for T21-only and Eup-only analyses to remove confounding differences among cell lines within each genotype. Sex was used for covariate correction in T21Unt and EupUnt comparisons, since male and female lines showed separation by MDS. Each candidate molecule was tested independently; thus, T21Unt versus EupUnt DEG datasets were generated for each candidate molecule analysis. To determine if candidate molecules corrected or worsened T21-associated gene dysregulation, the DEG list generated from the same experiment was compared with candidate molecule-induced DEGs.

Pathway analyses were performed using the ROSALIND knowledgebase, Metascape 3.5 (Zhou et al., 2019), or IPA (winter 2025 release, Qiagen).

## Supplementary Material

10.1242/biolopen.062807_sup1 Supplementary information

Table S1. Candidate pharmacotherapies tested for effects on growth of euploid and T21 NPCs.

Table S2. Euploid and T21 iPSCs and NPCs used in this study.

Table S3. Viability and inflammatory marker results in LPS-challenged RAW 264.7 cells and IMG cells.

Table S4. Comparison of gene expression in untreated T21 vs. untreated euploid NPCs.

Table S5. Comparison of gene expression in fasudil-treated T21 vs. untreated T21 NPCs.

Table S6. Identification of 1356 overlapping DEGs between T21Fas DEGs and T21 DEGs.

Table S7. Comparison of gene expression in fasudil-treated euploid vs. untreated euploid NPCs.

Table S8. Comparison of gene expression in EGCG-treated T21 vs. untreated T21 NPCs.

Table S9. Comparison of gene expression in sphingosine-treated T21 vs. untreated T21 NPCs.

Table S10. Comparison of gene expression in apigenin-treated T21 vs. untreated T21 NPCs and isoxsuprine-treated T21 vs. untreated T21 NPCs.

Table S11. Comparison of gene expression in EGCG-treated, apigenin-treated, sphingosine-treated, and isoxuprine-treated euploid NPCs vs. untreated euploid NPCs.

## References

[BIO062807C1] Abeysekera, I., Thomas, J., Georgiadis, T. M., Berman, A. G., Hammond, M. A., Dria, K. J., Wallace, J. M. and Roper, R. J. (2016). Differential effects of epigallocatechin-3-gallate containing supplements on correcting skeletal defects in a Down syndrome mouse model. *Mol. Nutr. Food Res.* 60, 717-726. 10.1002/mnfr.201500781 PMC4828301 26748562

[BIO062807C2] Alldred, M. J., Pidikiti, H., Heguy, A., Roussos, P. and Ginsberg, S. D. (2023). Basal forebrain cholinergic neurons are vulnerable in a mouse model of Down syndrome and their molecular fingerprint is rescued by maternal choline supplementation. *FASEB J.* 37, e22944. 10.1096/fj.202202111RR PMC10292934 37191946

[BIO062807C3] Alldred, M. J., Pidikiti, H., Ibrahim, K. W., Lee, S. H., Heguy, A., Chiosis, G., Mufson, E. J., Stutzmann, G. E. and Ginsberg, S. D. (2025). Benefits of maternal choline supplementation on aged basal forebrain cholinergic neurons (BFCNs) in a mouse model of Down syndrome and Alzheimer's disease. *Biomolecules* 15, 1131. 10.3390/biom15081131 PMC12384390 40867575

[BIO062807C4] Anderson, S. R., Jin, R. M. and Molofsky, A. V. (2025). Neuroimmune mechanisms of neurodevelopmental disorders. *Biol. Psychiatry* 99, 949-959. 10.1016/j.biopsych.2025.10.023 41173201

[BIO062807C5] Banerjee, A., Lu, Y., Do, K., Mize, T., Wu, X., Chen, X. and Chen, J. (2021). Validation of induced microglia-like cells (iMG cells) for future studies of brain diseases. *Front. Cell Neurosci.* 15, 629279. 10.3389/fncel.2021.629279 PMC8063054 33897370

[BIO062807C6] Belgacem, Y. H., Hamilton, A. M., Shim, S., Spencer, K. A. and Borodinsky, L. N. (2016). The many hats of Sonic Hedgehog signaling in nervous system development and disease. *J. Dev. Biol.* 4, 35. 10.3390/jdb4040035 PMC5831807 29615598

[BIO062807C7] Bianchi, P., Ciani, E., Guidi, S., Trazzi, S., Felice, D., Grossi, G., Fernandez, M., Giuliani, A., Calza, L. and Bartesaghi, R. (2010). Early pharmacotherapy restores neurogenesis and cognitive performance in the Ts65Dn mouse model for Down syndrome. *J. Neurosci.* 30, 8769-8779. 10.1523/JNEUROSCI.0534-10.2010 PMC6632890 20592198

[BIO062807C8] Blazek, J. D., Abeysekera, I., Li, J. and Roper, R. J. (2015). Rescue of the abnormal skeletal phenotype in Ts65Dn Down syndrome mice using genetic and therapeutic modulation of trisomic Dyrk1a. *Hum. Mol. Genet.* 24, 5687-5696. 10.1093/hmg/ddv284 26206885

[BIO062807C9] Briggs, J. A., Sun, J., Shepherd, J., Ovchinnikov, D. A., Chung, T. L., Nayler, S. P., Kao, L. P., Morrow, C. A., Thakar, N. Y., Soo, S. Y. et al. (2013). Integration-free induced pluripotent stem cells model genetic and neural developmental features of Down syndrome etiology. *Stem Cells* 31, 467-478. 10.1002/stem.1297 23225669

[BIO062807C10] Busciglio, J. and Yankner, B. A. (1995). Apoptosis and increased generation of reactive oxygen species in Down's syndrome neurons *in vitro*. *Nature* 378, 776-779. 10.1038/378776a0 8524410

[BIO062807C11] Butruille, L., Mayeur, S., Duparc, T., Knauf, C., Moitrot, E., Fajardy, I., Valet, P., Storme, L., Deruelle, P. and Lesage, J. (2012). Prenatal fasudil exposure alleviates fetal growth but programs hyperphagia and overweight in the adult male rat. *Eur. J. Pharmacol.* 689, 278-284. 10.1016/j.ejphar.2012.05.040 22683867

[BIO062807C12] Capasso, L., De Masi, L., Sirignano, C., Maresca, V., Basile, A., Nebbioso, A., Rigano, D. and Bontempo, P. (2025). Epigallocatechin gallate (EGCG): pharmacological properties, biological activities and therapeutic potential. *Molecules* 30, 654. 10.3390/molecules30030654 PMC11821029 39942757

[BIO062807C13] Catuara-Solarz, S., Espinosa-Carrasco, J., Erb, I., Langohr, K., Notredame, C., Gonzalez, J. R. and Dierssen, M. (2015). Principal component analysis of the effects of environmental enrichment and (−)-epigallocatechin-3-gallate on age-associated learning deficits in a mouse model of Down syndrome. *Front. Behav. Neurosci.* 9, 330. 10.3389/fnbeh.2015.00330 PMC4675859 26696850

[BIO062807C14] Chen, Y., Tristan, C. A., Chen, L., Jovanovic, V. M., Malley, C., Chu, P. H., Ryu, S., Deng, T., Ormanoglu, P., Tao, D. et al. (2021). A versatile polypharmacology platform promotes cytoprotection and viability of human pluripotent and differentiated cells. *Nat. Methods* 18, 528-541. 10.1038/s41592-021-01126-2 PMC8314867 33941937

[BIO062807C15] Cheng, Z., Erhardt, S. and Wang, J. (2025). Rho/ROCK pathway as a therapeutic target in multiple diseases. *Int. J. Drug Discov. Pharm.* 4, 10.53941/ijddp.2025.100018. 10.53941/ijddp.2025.100018 PMC12687578 41377066

[BIO062807C16] Cheon, S. Y., Kim, E. J., Kim, J. M., Kam, E. H., Ko, B. W. and Koo, B.-N. (2017). Regulation of microglia and macrophage polarization via apoptosis signal-regulating kinase 1 silencing after ischemic/hypoxic injury. *Front. Mol. Neurosci.* 10, 261. 10.3389/fnmol.2017.00261 PMC5557792 28855861

[BIO062807C17] Chi, C., Knight, W. E., Riching, A. S., Zhang, Z., Tatavosian, R., Zhuang, Y., Moldovan, R., Rachubinski, A. L., Gao, D., Xu, H. et al. (2023). Interferon hyperactivity impairs cardiogenesis in Down syndrome via downregulation of canonical Wnt signaling. *iScience* 26, 107012. 10.1016/j.isci.2023.107012 PMC10285545 37360690

[BIO062807C18] Cieuta-Walti, C., Cuenca-Royo, A., Langohr, K., Rakic, C., López-Vílchez, M. A., Lirio, J., González-Lamuño Leguina, D., González, T. B., García, J. G., Roure, M. R. et al. (2022). Safety and preliminary efficacy on cognitive performance and adaptive functionality of epigallocatechin gallate (EGCG) in children with Down syndrome. A randomized phase Ib clinical trial (PERSEUS study). *Genet. Med.* 24, 2004-2013. 10.1016/j.gim.2022.06.011 35951014

[BIO062807C19] Contestabile, A., Fila, T., Ceccarelli, C., Bonasoni, P., Bonapace, L., Santini, D., Bartesaghi, R. and Ciani, E. (2007). Cell cycle alteration and decreased cell proliferation in the hippocampal dentate gyrus and in the neocortical germinal matrix of fetuses with Down syndrome and in Ts65Dn mice. *Hippocampus* 17, 665-678. 10.1002/hipo.20308 17546680

[BIO062807C20] Das, I., Park, J. M., Shin, J. H., Jeon, S. K., Lorenzi, H., Linden, D. J., Worley, P. F. and Reeves, R. H. (2013). Hedgehog agonist therapy corrects structural and cognitive deficits in a Down syndrome mouse model. *Sci. Transl. Med.* 5, 201ra120. 10.1126/scitranslmed.3005983 PMC4006719 24005160

[BIO062807C21] Davisson, M. T., Schmidt, C. and Akeson, E. C. (1990). Segmental trisomy of murine chromosome 16: a new model system for studying Down syndrome. *Prog. Clin. Biol. Res.* 360, 263-280. 2147289

[BIO062807C22] De la Torre, R., De Sola, S., Pons, M., Duchon, A., de Lagran, M. M., Farré, M., Fitó, M., Benejam, B., Langohr, K., Rodriguez, J. et al. (2014). Epigallocatechin-3-gallate, a DYRK1A inhibitor, rescues cognitive deficits in Down syndrome mouse models and in humans. *Mol. Nutr. Food Res.* 58, 278-288. 10.1002/mnfr.201300325 24039182

[BIO062807C23] de la Torre, R., de Sola, S., Hernandez, G., Farre, M., Pujol, J., Rodriguez, J., Espadaler, J. M., Langohr, K., Cuenca-Royo, A., Principe, A. et al. (2016). Safety and efficacy of cognitive training plus epigallocatechin-3-gallate in young adults with Down's syndrome (TESDAD): a double-blind, randomised, placebo-controlled, phase 2 trial. *Lancet Neurol.* 15, 801-810. 10.1016/S1474-4422(16)30034-5 27302362

[BIO062807C24] Duchon, A., Raveau, M., Chevalier, C., Nalesso, V., Sharp, A. J. and Herault, Y. (2011). Identification of the translocation breakpoints in the Ts65Dn and Ts1Cje mouse lines: relevance for modeling Down syndrome. *Mamm. Genome* 22, 674-684. 10.1007/s00335-011-9356-0 PMC3224224 21953411

[BIO062807C25] Feng, Y., LoGrasso, P. V., Defert, O. and Li, R. (2016). Rho Kinase (ROCK) inhibitors and their therapeutic potential. *J. Med. Chem.* 59, 2269-2300. 10.1021/acs.jmedchem.5b00683 26486225

[BIO062807C26] Flores-Aguilar, L., Iulita, M. F., Kovecses, O., Torres, M. D., Levi, S. M., Zhang, Y., Askenazi, M., Wisniewski, T., Busciglio, J. and Cuello, A. C. (2020). Evolution of neuroinflammation across the lifespan of individuals with Down syndrome. *Brain* 143, 3653-3671. 10.1093/brain/awaa326 PMC7805813 33206953

[BIO062807C27] Galbraith, M. D., Rachubinski, A. L., Smith, K. P., Araya, P., Waugh, K. A., Enriquez-Estrada, B., Worek, K., Granrath, R. E., Kinning, K. T., Paul Eduthan, N. et al. (2023). Multidimensional definition of the interferonopathy of Down syndrome and its response to JAK inhibition. *Sci. Adv.* 9, eadg6218. 10.1126/sciadv.adg6218 PMC10306300 37379383

[BIO062807C28] Gao, F. J., Klinedinst, D., Fernandez, F. X., Cheng, B., Savonenko, A., Devenney, B., Li, Y., Wu, D., Pomper, M. G. and Reeves, R. H. (2021). Forebrain Shh overexpression improves cognitive function and locomotor hyperactivity in an aneuploid mouse model of Down syndrome and its euploid littermates. *Acta Neuropathol. Commun.* 9, 137. 10.1186/s40478-021-01237-z PMC8365939 34399854

[BIO062807C29] García-Cerro, S., Rueda, N., Vidal, V., Puente, A., Campa, V., Lantigua, S., Narcís, O., Velasco, A., Bartesaghi, R. and Martínez-Cué, C. (2020). Prenatal administration of oleic acid or linolenic acid reduces neuromorphological and cognitive alterations in Ts65dn Down syndrome mice. *J. Nutr.* 150, 1631-1643. 10.1093/jn/nxaa074 32243527

[BIO062807C30] Gautier, M. K., Kelley, C. M., Lee, S. H., Alldred, M. J., McDaid, J., Mufson, E. J., Stutzmann, G. E. and Ginsberg, S. D. (2023). Maternal choline supplementation protects against age-associated cholinergic and GABAergic basal forebrain neuron degeneration in the Ts65Dn mouse model of Down syndrome and Alzheimer's disease. *Neurobiol. Dis.* 188, 106332. 10.1016/j.nbd.2023.106332 PMC10752300 37890559

[BIO062807C31] Gautier, M. K., Kelley, C. M., Lee, S. H., Mufson, E. J. and Ginsberg, S. D. (2024). Maternal choline supplementation rescues early endosome pathology in basal forebrain cholinergic neurons in the Ts65Dn mouse model of Down syndrome and Alzheimer's disease. *Neurobiol. Aging* 144, 30-42. 10.1016/j.neurobiolaging.2024.09.002 PMC11490376 39265450

[BIO062807C32] Ghasemi-Tarie, R., Kiasalari, Z., Fakour, M., Khorasani, M., Keshtkar, S., Baluchnejadmojarad, T. and Roghani, M. (2022). Nobiletin prevents amyloid beta(1-40)-induced cognitive impairment via inhibition of neuroinflammation and oxidative/nitrosative stress. *Metab. Brain Dis.* 37, 1337-1349. 10.1007/s11011-022-00949-y 35294678

[BIO062807C33] Giacomini, A., Stagni, F., Emili, M., Uguagliati, B., Rimondini, R., Bartesaghi, R. and Guidi, S. (2019). Timing of treatment with the flavonoid 7,8-DHF critically impacts on its effects on learning and memory in the Ts65Dn mouse. *Antioxidants (Basel)* 8, 163. 10.3390/antiox8060163 PMC6617346 31174258

[BIO062807C34] Glotfelty, E. J., Tovar-Y-Romo, L. B., Hsueh, S.-C., Tweedie, D., Li, Y., Harvey, B. K., Hoffer, B. J., Karlsson, T. E., Olson, L. and Greig, N. H. (2023). The RhoA-ROCK1/ROCK2 pathway exacerbates inflammatory signaling in immortalized and primary microglia. *Cells* 12, 1367. 10.3390/cells12101367 PMC10216802 37408199

[BIO062807C35] Gomez-Larrauri, A., Larrea-Sebal, A., Martín, C. and Gomez-Muñoz, A. (2025). The critical roles of bioactive sphingolipids in inflammation. *J. Biol. Chem.* 301, 110475. 10.1016/j.jbc.2025.110475 PMC12340401 40653199

[BIO062807C36] Goodlett, C. R., Stringer, M., LaCombe, J., Patel, R., Wallace, J. M. and Roper, R. J. (2020). Evaluation of the therapeutic potential of epigallocatechin-3-gallate (EGCG) via oral gavage in young adult Down syndrome mice. *Sci. Rep.* 10, 10426. 10.1038/s41598-020-67133-z PMC7319987 32591597

[BIO062807C37] Gu, Y., Feng, Y., Yu, J., Yuan, H., Yin, Y., Ding, J., Zhao, J., Xu, Y., Xu, J. and Che, H. (2017). Fasudil attenuates soluble fms-like tyrosine kinase-1 (sFlt-1)-induced hypertension in pregnant mice through RhoA/ROCK pathway. *Oncotarget* 8, 104104-104112. 10.18632/oncotarget.22017 PMC5732790 29262624

[BIO062807C38] Guedj, F., Bianchi, D. W. and Delabar, J. M. (2014). Prenatal treatment of Down syndrome: a reality? *Curr. Opin. Obstet. Gynecol.* 26, 92-103. 10.1097/GCO.0000000000000056 24573065

[BIO062807C39] Guedj, F., Pennings, J. L., Massingham, L. J., Wick, H. C., Siegel, A. E., Tantravahi, U. and Bianchi, D. W. (2016). An integrated human/murine transcriptome and pathway approach to identify prenatal treatments for Down syndrome. *Sci. Rep.* 6, 32353. 10.1038/srep32353 PMC5009456 27586445

[BIO062807C40] Guedj, F., Siegel, A. E., Pennings, J. L. A., Alsebaa, F., Massingham, L. J., Tantravahi, U. and Bianchi, D. W. (2020). Apigenin as a candidate prenatal treatment for trisomy 21: effects in human amniocytes and the Ts1Cje mouse model. *Am. J. Hum. Genet.* 107, 911-931. 10.1016/j.ajhg.2020.10.001 PMC7675036 33098770

[BIO062807C41] Guedj, F., Kane, E., Bishop, L. A., Pennings, J. L. A., Herault, Y. and Bianchi, D. W. (2023). The impact of Mmu17 non-Hsa21 orthologous genes in the Ts65Dn mouse model of Down syndrome: the gold standard refuted. *Biol. Psychiatry* 94, 84-97. 10.1016/j.biopsych.2023.02.012 PMC10330375 37074246

[BIO062807C42] Guidi, S., Bonasoni, P., Ceccarelli, C., Santini, D., Gualtieri, F., Ciani, E. and Bartesaghi, R. (2008). Neurogenesis impairment and increased cell death reduce total neuron number in the hippocampal region of fetuses with Down syndrome. *Brain Pathol.* 18, 180-197. 10.1111/j.1750-3639.2007.00113.x PMC8095525 18093248

[BIO062807C43] Guidi, S., Stagni, F., Bianchi, P., Ciani, E., Ragazzi, E., Trazzi, S., Grossi, G., Mangano, C., Calzà, L. and Bartesaghi, R. (2013). Early pharmacotherapy with fluoxetine rescues dendritic pathology in the Ts65Dn mouse model of Down syndrome. *Brain Pathol.* 23, 129-143. 10.1111/j.1750-3639.2012.00624.x PMC8028975 22817700

[BIO062807C44] Guidi, S., Stagni, F., Bianchi, P., Ciani, E., Giacomini, A., De Franceschi, M., Moldrich, R., Kurniawan, N., Mardon, K., Giuliani, A. et al. (2014). Prenatal pharmacotherapy rescues brain development in a Down's syndrome mouse model. *Brain* 137, 380-401. 10.1093/brain/awt340 24334313

[BIO062807C45] Guidi, S., Giacomini, A., Stagni, F., Emili, M., Uguagliati, B., Bonasoni, M. P. and Bartesaghi, R. (2018). Abnormal development of the inferior temporal region in fetuses with Down syndrome. *Brain Pathol.* 28, 986-998. 10.1111/bpa.12605 PMC8028380 29509279

[BIO062807C46] Guo, M.-F., Zhang, H.-Y., Li, Y.-H., Gu, Q.-F., Wei, W.-Y., Wang, Y.-Y., Zhang, X.-J., Liu, X.-Q., Song, L.-J., Chai, Z. et al. (2020). Fasudil inhibits the activation of microglia and astrocytes of transgenic Alzheimer's disease mice via the downregulation of TLR4/Myd88/NF-kappaB pathway. *J. Neuroimmunol.* 346, 577284. 10.1016/j.jneuroim.2020.577284 32652366

[BIO062807C47] Heinen, M., Hettich, M. M., Ryan, D. P., Schnell, S., Paesler, K. and Ehninger, D. (2012). Adult-onset fluoxetine treatment does not improve behavioral impairments and may have adverse effects on the Ts65Dn mouse model of Down syndrome. *Neural Plast.* 2012, 467251. 10.1155/2012/467251 PMC3405721 22848851

[BIO062807C48] Hou, S.-W., Liu, C.-Y., Li, Y.-H., Yu, J.-Z., Feng, L., Liu, Y.-T., Guo, M.-F., Xie, Y., Meng, J., Zhang, H.-F. et al. (2012). Fasudil ameliorates disease progression in experimental autoimmune encephalomyelitis, acting possibly through antiinflammatory effect. *CNS Neurosci. Ther.* 18, 909-917. 10.1111/cns.12002 PMC6493591 22994384

[BIO062807C49] Hsueh, S. C., Parekh, P., Batsaikhan, B., Vargesson, N., Tweedie, D., Luo, W., Patel, C. N., Liu, D., McDevitt, R. A., Baig, A. M. et al. (2025). Targeting neuroinflammation: 3-monothiopomalidomide a new drug candidate to mitigate traumatic brain injury and neurodegeneration. *J. Biomed. Sci.* 32, 57. 10.1186/s12929-025-01150-w PMC12172326 40524167

[BIO062807C50] Huang, T., Fakurazi, S., Cheah, P. S. and Ling, K. H. (2025). Dysregulation of REST and its target genes impacts the fate of neural progenitor cells in Down syndrome. *Sci. Rep.* 15, 2818. 10.1038/s41598-025-87314-y PMC11754635 39843579

[BIO062807C51] Hwang, S., Williams, J. F., Kneissig, M., Lioudyno, M., Rivera, I., Helguera, P., Busciglio, J., Storchova, Z., King, M. C. and Torres, E. M. (2019). Suppressing aneuploidy-associated phenotypes improves the fitness of trisomy 21 cells. *Cell Rep.* 29, 2473-2488.e2475. 10.1016/j.celrep.2019.10.059 PMC6886690 31747614

[BIO062807C52] Izzo, A., Nitti, M., Mollo, N., Paladino, S., Procaccini, C., Faicchia, D., Calì, G., Genesio, R., Bonfiglio, F., Cicatiello, R. et al. (2017). Metformin restores the mitochondrial network and reverses mitochondrial dysfunction in Down syndrome cells. *Hum. Mol. Genet.* 26, 1056-1069. 10.1093/hmg/ddx016 28087733

[BIO062807C53] Jahan, S., Ansari, U. A., Srivastava, A. K., Aldosari, S., Alabdallat, N. G., Siddiqui, A. J., Khan, A., Albadrani, H. M., Sarkar, S., Khan, B. et al. (2024). A protein-miRNA biomic analysis approach to explore neuroprotective potential of nobiletin in human neural progenitor cells (hNPCs). *Front. Pharmacol.* 15, 1343569. 10.3389/fphar.2024.1343569 PMC10860404 38348393

[BIO062807C54] Jamal, R., LaCombe, J., Patel, R., Blackwell, M., Thomas, J. R., Sloan, K., Wallace, J. M. and Roper, R. J. (2022). Increased dosage and treatment time of epigallocatechin-3-gallate (EGCG) negatively affects skeletal parameters in normal mice and Down syndrome mouse models. *PLoS ONE* 17, e0264254. 10.1371/journal.pone.0264254 PMC8865638 35196359

[BIO062807C55] Jin, M., Xu, R., Wang, L., Alam, M. M., Ma, Z., Zhu, S., Martini, A. C., Jadali, A., Bernabucci, M., Xie, P. et al. (2022). Type-I-interferon signaling drives microglial dysfunction and senescence in human iPSC models of Down syndrome and Alzheimer's disease. *Cell Stem Cell* 29, 1135-1153.e1138. 10.1016/j.stem.2022.06.007 PMC9345168 35803230

[BIO062807C56] Keenan, A. B., Jenkins, S. L., Jagodnik, K. M., Koplev, S., He, E., Torre, D., Wang, Z., Dohlman, A. B., Silverstein, M. C., Lachmann, A. et al. (2018). The Library of Integrated Network-Based Cellular Signatures NIH program: system-level cataloging of human cells response to perturbations. *Cell Syst.* 6, 13-24. 10.1016/j.cels.2017.11.001 PMC5799026 29199020

[BIO062807C57] Kitano, R., Madan, N., Mikami, T., Madankumar, R., Skotko, B. G., Santoro, S., Ralston, S. J., Bianchi, D. W. and Tarui, T. (2023). Biometric magnetic resonance imaging analysis of fetal brain development in Down syndrome. *Prenat. Diagn.* 43, 1450-1458. 10.1002/pd.6436 PMC11742279 37698481

[BIO062807C58] Klein, J. A. and Haydar, T. F. (2022). Neurodevelopment in Down syndrome: concordance in humans and models. *Front. Cell Neurosci.* 16, 941855. 10.3389/fncel.2022.941855 PMC9334873 35910249

[BIO062807C59] Koch, J. C., Tatenhorst, L., Roser, A.-E., Saal, K.-A., Tönges, L. and Lingor, P. (2018). ROCK inhibition in models of neurodegeneration and its potential for clinical translation. *Pharmacol. Ther.* 189, 1-21. 10.1016/j.pharmthera.2018.03.008 29621594

[BIO062807C60] Koch, J. C., Leha, A., Bidner, H., Cordts, I., Dorst, J., Günther, R., Zeller, D., Braun, N., Metelmann, M., Corcia, P. et al. (2024). Safety, tolerability, and efficacy of fasudil in amyotrophic lateral sclerosis (ROCK-ALS): a phase 2, randomised, double-blind, placebo-controlled trial. *Lancet Neurol.* 23, 1133-1146. 10.1016/S1474-4422(24)00373-9 PMC12741558 39424560

[BIO062807C61] Kopp, K. O., Li, Y., Glotfelty, E. J., Tweedie, D. and Greig, N. H. (2024). Incretin-based multi-agonist peptides are neuroprotective and anti-inflammatory in cellular models of neurodegeneration. *Biomolecules* 14, 872. 10.3390/biom14070872 PMC11275108 39062586

[BIO062807C62] Lamb, J., Crawford, E. D., Peck, D., Modell, J. W., Blat, I. C., Wrobel, M. J., Lerner, J., Brunet, J.-P., Subramanian, A., Ross, K. N. et al. (2006). The Connectivity Map: using gene-expression signatures to connect small molecules, genes, and disease. *Science* 313, 1929-1935. 10.1126/science.1132939 17008526

[BIO062807C63] Lawrence, A. R., Canzi, A., Bridlance, C., Olivié, N., Lansonneur, C., Catale, C., Pizzamiglio, L., Kloeckner, B., Silvin, A., Munro, D. A. D. et al. (2024). Microglia maintain structural integrity during fetal brain morphogenesis. *Cell* 187, 962-980.e919. 10.1016/j.cell.2024.01.012 PMC10869139 38309258

[BIO062807C64] LeBlanc-Straceski, J., Williams, R., Ward, K., Bates, A., Duran, C., Anderson, L., Murray, M., Pereira-Badji, J., Shoushani, C. and Thibault, J. (2022). ROCK2 knockout improves proliferation rate in a cellular model of Down syndrome. *bioRxiv*. 10.1101/2022.10.20.513071

[BIO062807C65] Lecca, D., Jung, Y. J., Scerba, M. T., Hwang, I., Kim, Y. K., Kim, S., Modrow, S., Tweedie, D., Hsueh, S.-C., Liu, D. et al. (2022). Role of chronic neuroinflammation in neuroplasticity and cognitive function: a hypothesis. *Alzheimers Dement.* 18, 2327-2340. 10.1002/alz.12610 PMC9437140 35234334

[BIO062807C66] Lee, S. E., Baxter, L. L., Duran, M. I., Morris, S. D., Mosley, I. A., Fuentes, K. A., Pennings, J. L. A., Guedj, F. and Bianchi, D. W. (2025). Analysis of genotype effects and inter-individual variability in iPSC-derived trisomy 21 neural progenitor cells. *Hum. Mol. Genet.* 34, 85-100. 10.1093/hmg/ddae160 PMC12034096 39533854

[BIO062807C67] Lewanda, A. F., Gallegos, M. F. and Summar, M. (2018). Patterns of dietary supplement use in children with Down syndrome. *J. Pediatr.* 201, 100-105.e130. 10.1016/j.jpeds.2018.05.022 29961644

[BIO062807C68] Li, Y., Glotfelty, E. J., Karlsson, T., Fortuno, L. V., Harvey, B. K. and Greig, N. H. (2021). The metabolite GLP-1 (9-36) is neuroprotective and anti-inflammatory in cellular models of neurodegeneration. *J. Neurochem.* 159, 867-886. 10.1111/jnc.15521 PMC8627453 34569615

[BIO062807C69] Liu, H., Pan, Z., Ma, X., Cui, J., Gao, J., Miao, Q., Zhu, Z., Chen, X. and Su, S. (2022). ROCK inhibitor fasudil reduces the expression of inflammatory factors in LPS-induced rat pulmonary microvascular endothelial cells via ROS/NF-kappaB pathway. *BMC Pharmacol. Toxicol.* 23, 24. 10.1186/s40360-022-00565-7 PMC9013060 35428330

[BIO062807C70] López-Hidalgo, R., Ballestin, R., Lorenzo, L., Sánchez-Martí, S., Blasco-Ibáñez, J. M., Crespo, C., Nacher, J. and Varea, E. (2024). Early chronic fasudil treatment rescues hippocampal alterations in the Ts65Dn model for Down syndrome. *Neurochem. Int.* 174, 105679. 10.1016/j.neuint.2024.105679 38309665

[BIO062807C71] Lu, J., Lian, G., Zhou, H., Esposito, G., Steardo, L., Delli-Bovi, L. C., Hecht, J. L., Lu, Q. R. and Sheen, V. (2012). OLIG2 over-expression impairs proliferation of human Down syndrome neural progenitors. *Hum. Mol. Genet.* 21, 2330-2340. 10.1093/hmg/dds052 PMC3335315 22343408

[BIO062807C72] Martinez-Cue, C. and Bartesaghi, R. (2022). Fatty acids: a safe tool for improving neurodevelopmental alterations in Down syndrome? *Nutrients* 14, 2880. 10.3390/nu14142880 PMC9323400 35889838

[BIO062807C73] Martini, A. C., Helman, A. M., McCarty, K. L., Lott, I. T., Doran, E., Schmitt, F. A. and Head, E. (2020). Distribution of microglial phenotypes as a function of age and Alzheimer's disease neuropathology in the brains of people with Down syndrome. *Alzheimers Dement. (Amst)* 12, e12113. 10.1002/dad2.12113 PMC7560512 33088896

[BIO062807C74] McElyea, S. D., Starbuck, J. M., Tumbleson-Brink, D. M., Harrington, E., Blazek, J. D., Ghoneima, A., Kula, K. and Roper, R. J. (2016). Influence of prenatal EGCG treatment and Dyrk1a dosage reduction on craniofacial features associated with Down syndrome. *Hum. Mol. Genet.* 25, 4856-4869. 10.1093/hmg/ddw309 PMC6049609 28172997

[BIO062807C75] Meharena, H. S., Marco, A., Dileep, V., Lockshin, E. R., Akatsu, G. Y., Mullahoo, J., Watson, L. A., Ko, T., Guerin, L. N., Abdurrob, F. et al. (2022). Down-syndrome-induced senescence disrupts the nuclear architecture of neural progenitors. *Cell Stem Cell* 29, 116-130.e117. 10.1016/j.stem.2021.12.002 PMC8805993 34995493

[BIO062807C76] Mendiola, A. S., Yan, Z., Dixit, K., Johnson, J. R., Bouhaddou, M., Meyer-Franke, A., Shin, M.-G., Yong, Y., Agrawal, A., MacDonald, E. et al. (2023). Defining blood-induced microglia functions in neurodegeneration through multiomic profiling. *Nat. Immunol.* 24, 1173-1187. 10.1038/s41590-023-01522-0 PMC10307624 37291385

[BIO062807C77] Mollo, N., Nitti, M., Zerillo, L., Faicchia, D., Micillo, T., Accarino, R., Secondo, A., Petrozziello, T., Calì, G., Cicatiello, R. et al. (2019). Pioglitazone improves mitochondrial organization and bioenergetics in Down syndrome cells. *Front. Genet.* 10, 606. 10.3389/fgene.2019.00606 PMC6609571 31316549

[BIO062807C78] Moon, J., Chen, M., Gandhy, S. U., Strawderman, M., Levitsky, D. A., Maclean, K. N. and Strupp, B. J. (2010). Perinatal choline supplementation improves cognitive functioning and emotion regulation in the Ts65Dn mouse model of Down syndrome. *Behav. Neurosci.* 124, 346-361. 10.1037/a0019590 PMC2955960 20528079

[BIO062807C79] Nakajima, A. and Ohizumi, Y. (2019). Potential benefits of nobiletin, a citrus flavonoid, against Alzheimer's Disease and Parkinson's disease. *Int. J. Mol. Sci.* 20, 3380. 10.3390/ijms20143380 PMC6678479 31295812

[BIO062807C80] Nouri, Z., Fakhri, S., El-Senduny, F. F., Sanadgol, N., Abd-ElGhani, G. E., Farzaei, M. H. and Chen, J. T. (2019). On the neuroprotective effects of naringenin: pharmacological targets, signaling pathways, molecular mechanisms, and clinical perspective. *Biomolecules* 9, 690. 10.3390/biom9110690 PMC6920995 31684142

[BIO062807C81] Okamoto, H., Yoshio, T., Kaneko, H. and Yamanaka, H. (2010). Inhibition of NF-kappaB signaling by fasudil as a potential therapeutic strategy for rheumatoid arthritis. *Arthritis. Rheum.* 62, 82-92. 10.1002/art.25063 20039418

[BIO062807C82] Pinto, B., Morelli, G., Rastogi, M., Savardi, A., Fumagalli, A., Petretto, A., Bartolucci, M., Varea, E., Catelani, T., Contestabile, A. et al. (2020). Rescuing over-activated microglia restores cognitive performance in juvenile animals of the Dp(16) mouse model of Down syndrome. *Neuron* 108, 887-904.e812. 10.1016/j.neuron.2020.09.010 PMC7736620 33027640

[BIO062807C83] Pooh, R. K., Machida, M., Uenishi, K., Barreto, E. Q. S., Man Wah, I. Y., Poon, L. C., Itoh, K., Nakamura, T., Chiyo, H., Ohashi, H. et al. (2025). The fetal brain neurosonography in trisomy 21: the seagull sign and thinned subplate. *Am. J. Obstet. Gynecol.* 233, 315.e311-315.e320. 10.1016/j.ajog.2025.04.054 40311828

[BIO062807C84] Powers, B. E., Kelley, C. M., Velazquez, R., Ash, J. A., Strawderman, M. S., Alldred, M. J., Ginsberg, S. D., Mufson, E. J. and Strupp, B. J. (2017). Maternal choline supplementation in a mouse model of Down syndrome: effects on attention and nucleus basalis/substantia innominata neuron morphology in adult offspring. *Neuroscience* 340, 501-514. 10.1016/j.neuroscience.2016.11.001 PMC5177989 27840230

[BIO062807C85] Powers, B. E., Velazquez, R., Strawderman, M. S., Ginsberg, S. D., Mufson, E. J. and Strupp, B. J. (2021). Maternal choline supplementation as a potential therapy for Down syndrome: assessment of effects throughout the lifespan. *Front. Aging Neurosci.* 13, 723046. 10.3389/fnagi.2021.723046 PMC8527982 34690739

[BIO062807C86] Rachubinski, A. L., Wallace, E., Gurnee, E., Enriquez-Estrada, B. A., Worek, K. R., Smith, K. P., Araya, P., Waugh, K. A., Granrath, R. E., Britton, E. et al. (2024). JAK inhibition decreases the autoimmune burden in Down syndrome. *eLife* 13, RP99323. 10.7554/eLife.99323 PMC11687933 39737640

[BIO062807C87] Reeves, R. H., Irving, N. G., Moran, T. H., Wohn, A., Kitt, C., Sisodia, S. S., Schmidt, C., Bronson, R. T. and Davisson, M. T. (1995). A mouse model for Down syndrome exhibits learning and behaviour deficits. *Nat. Genet.* 11, 177-184. 10.1038/ng1095-177 7550346

[BIO062807C88] Risgaard, R. D., Hanthanan Arachchilage, K., Knaack, S. A., Hosseini, M., Chen, R. J., Kumarage, P., Schmidt, D. K., Huang, X., Sheng, J., Wang, C. J. et al. (2026). Molecular and cellular processes disrupted in the early postnatal Down syndrome prefrontal cortex. *Science* 392, eaea1549. 10.1126/science.aea1549 PMC13286256 42024756

[BIO062807C89] Roper, R. J., Baxter, L. L., Saran, N. G., Klinedinst, D. K., Beachy, P. A. and Reeves, R. H. (2006). Defective cerebellar response to mitogenic Hedgehog signaling in Down [corrected] syndrome mice. *Proc. Natl. Acad. Sci. USA* 103, 1452-1456. 10.1073/pnas.0510750103 PMC1360600 16432181

[BIO062807C90] Roser, A. E., Tönges, L. and Lingor, P. (2017). Modulation of microglial activity by Rho-Kinase (ROCK) inhibition as therapeutic strategy in Parkinson's disease and amyotrophic lateral sclerosis. *Front. Aging Neurosci* 9, 94. 10.3389/fnagi.2017.00094 PMC5378706 28420986

[BIO062807C91] Roskoski, R. Jr. (2023). Small molecule protein kinase inhibitors approved by regulatory agencies outside of the United States. *Pharmacol. Res.* 194, 106847. 10.1016/j.phrs.2023.106847 37454916

[BIO062807C92] Rueda, N., Flórez, J., Dierssen, M. and Martínez-Cué, C. (2020a). Translational validity and implications of pharmacotherapies in preclinical models of Down syndrome. *Prog. Brain Res.* 251, 245-268. 10.1016/bs.pbr.2019.10.001 32057309

[BIO062807C93] Rueda, N., Vidal, V., Garcia-Cerro, S., Puente, A., Campa, V., Lantigua, S., Narcís, O., Bartesaghi, R. and Martínez-Cué, C. (2020b). Prenatal, but not postnatal, curcumin administration rescues neuromorphological and cognitive alterations in Ts65Dn Down syndrome mice. *J. Nutr.* 150, 2478-2489. 10.1093/jn/nxaa207 32729926

[BIO062807C94] Russo, M. L., Sousa, A. M. M. and Bhattacharyya, A. (2024). Consequences of trisomy 21 for brain development in Down syndrome. *Nat. Rev. Neurosci.* 25, 740-755. 10.1038/s41583-024-00866-2 PMC11834940 39379691

[BIO062807C95] Sago, H., Carlson, E. J., Smith, D. J., Kilbridge, J., Rubin, E. M., Mobley, W. C., Epstein, C. J. and Huang, T. T. (1998). Ts1Cje, a partial trisomy 16 mouse model for Down syndrome, exhibits learning and behavioral abnormalities. *Proc. Natl. Acad. Sci. USA* 95, 6256-6261. 10.1073/pnas.95.11.6256 PMC27649 9600952

[BIO062807C96] Sapashnik, D., Newman, R., Pietras, C. M., Zhou, D., Devkota, K., Qu, F., Kofman, L., Boudreau, S., Fried, I. and Slonim, D. K. (2023). Cell-specific imputation of drug connectivity mapping with incomplete data. *PLoS ONE* 18, e0278289. 10.1371/journal.pone.0278289 PMC9934325 36795645

[BIO062807C97] Shi, J. and Wei, L. (2022). Rho kinases in embryonic development and stem cell research. *Arch. Immunol. Ther. Exp. (Warsz)* 70, 4. 10.1007/s00005-022-00642-z PMC8766376 35043239

[BIO062807C98] Shibuya, M., Suzuki, Y., Sugita, K., Saito, I., Sasaki, T., Takakura, K., Nagata, I., Kikuchi, H., Takemae, T., Hidaka, H. et al. (1992). Effect of AT877 on cerebral vasospasm after aneurysmal subarachnoid hemorrhage. Results of a prospective placebo-controlled double-blind trial. *J. Neurosurg.* 76, 571-577. 10.3171/jns.1992.76.4.0571 1545249

[BIO062807C99] Song, Y., Chen, X., Wang, L.-Y., Gao, W. and Zhu, M.-J. (2013). Rho kinase inhibitor fasudil protects against beta-amyloid-induced hippocampal neurodegeneration in rats. *CNS Neurosci. Ther.* 19, 603-610. 10.1111/cns.12116 PMC6493451 23638992

[BIO062807C100] Souchet, B., Duchon, A., Gu, Y., Dairou, J., Chevalier, C., Daubigney, F., Nalesso, V., Créau, N., Yu, Y., Janel, N. et al. (2019). Prenatal treatment with EGCG enriched green tea extract rescues GAD67 related developmental and cognitive defects in Down syndrome mouse models. *Sci. Rep.* 9, 3914. 10.1038/s41598-019-40328-9 PMC6408590 30850713

[BIO062807C101] Stagni, F. and Bartesaghi, R. (2022). The challenging pathway of treatment for neurogenesis impairment in Down syndrome: achievements and perspectives. *Front. Cell Neurosci.* 16, 903729. 10.3389/fncel.2022.903729 PMC9130961 35634470

[BIO062807C102] Stagni, F., Magistretti, J., Guidi, S., Ciani, E., Mangano, C., Calzà, L. and Bartesaghi, R. (2013). Pharmacotherapy with fluoxetine restores functional connectivity from the dentate gyrus to field CA3 in the Ts65Dn mouse model of Down syndrome. *PLoS ONE* 8, e61689. 10.1371/journal.pone.0061689 PMC3631158 23620781

[BIO062807C103] Stagni, F., Giacomini, A., Guidi, S., Ciani, E. and Bartesaghi, R. (2015a). Timing of therapies for Down syndrome: the sooner, the better. *Front. Behav. Neurosci.* 9, 265. 10.3389/fnbeh.2015.00265 PMC4594009 26500515

[BIO062807C104] Stagni, F., Giacomini, A., Guidi, S., Ciani, E., Ragazzi, E., Filonzi, M., De Iasio, R., Rimondini, R. and Bartesaghi, R. (2015b). Long-term effects of neonatal treatment with fluoxetine on cognitive performance in Ts65Dn mice. *Neurobiol. Dis.* 74, 204-218. 10.1016/j.nbd.2014.12.005 25497735

[BIO062807C105] Stagni, F., Giacomini, A., Emili, M., Trazzi, S., Guidi, S., Sassi, M., Ciani, E., Rimondini, R. and Bartesaghi, R. (2016). Short- and long-term effects of neonatal pharmacotherapy with epigallocatechin-3-gallate on hippocampal development in the Ts65Dn mouse model of Down syndrome. *Neuroscience* 333, 277-301. 10.1016/j.neuroscience.2016.07.031 27457036

[BIO062807C106] Stagni, F., Giacomini, A., Guidi, S., Emili, M., Uguagliati, B., Salvalai, M. E., Bortolotto, V., Grilli, M., Rimondini, R. and Bartesaghi, R. (2017). A flavonoid agonist of the TrkB receptor for BDNF improves hippocampal neurogenesis and hippocampus-dependent memory in the Ts65Dn mouse model of DS. *Exp. Neurol.* 298, 79-96. 10.1016/j.expneurol.2017.08.018 28882412

[BIO062807C107] Stagni, F., Giacomini, A., Emili, M., Guidi, S. and Bartesaghi, R. (2018). Neurogenesis impairment: An early developmental defect in Down syndrome. *Free Radic. Biol. Med.* 114, 15-32. 10.1016/j.freeradbiomed.2017.07.026 28756311

[BIO062807C108] Stagni, F., Giacomini, A., Emili, M., Uguagliati, B., Bonasoni, M. P., Bartesaghi, R. and Guidi, S. (2019). Subicular hypotrophy in fetuses with Down syndrome and in the Ts65Dn model of Down syndrome. *Brain Pathol.* 29, 366-379. 10.1111/bpa.12663 PMC8028278 30325080

[BIO062807C109] Stagni, F., Giacomini, A., Emili, M., Uguagliati, B., Bonasoni, M. P., Bartesaghi, R. and Guidi, S. (2020). Neuroanatomical alterations in higher-order thalamic nuclei of fetuses with Down syndrome. *Clin. Neurol. Neurosurg.* 194, 105870. 10.1016/j.clineuro.2020.105870 32480293

[BIO062807C110] Stagni, F., Uguagliati, B., Emili, M., Giacomini, A., Bartesaghi, R. and Guidi, S. (2021). The flavonoid 7,8-DHF fosters prenatal brain proliferation potency in a mouse model of Down syndrome. *Sci. Rep.* 11, 6300. 10.1038/s41598-021-85284-5 PMC7973813 33737521

[BIO062807C111] Starbuck, J. M., Llambrich, S., Gonzàlez, R., Albaigès, J., Sarlé, A., Wouters, J., González, A., Sevillano, X., Sharpe, J., De La Torre, R. et al. (2021). Green tea extracts containing epigallocatechin-3-gallate modulate facial development in Down syndrome. *Sci. Rep.* 11, 4715. 10.1038/s41598-021-83757-1 PMC7907288 33633179

[BIO062807C112] Stringer, M., Abeysekera, I., Thomas, J., LaCombe, J., Stancombe, K., Stewart, R. J., Dria, K. J., Wallace, J. M., Goodlett, C. R. and Roper, R. J. (2017). Epigallocatechin-3-gallate (EGCG) consumption in the Ts65Dn model of Down syndrome fails to improve behavioral deficits and is detrimental to skeletal phenotypes. *Physiol. Behav.* 177, 230-241. 10.1016/j.physbeh.2017.05.003 PMC5525541 28478033

[BIO062807C113] Subramanian, A., Narayan, R., Corsello, S. M., Peck, D. D., Natoli, T. E., Lu, X., Gould, J., Davis, J. F., Tubelli, A. A., Asiedu, J. K. et al. (2017). A next generation connectivity map: L1000 platform and the first 1,000,000 profiles. *Cell* 171, 1437-1452.e1417. 10.1016/j.cell.2017.10.049 PMC5990023 29195078

[BIO062807C114] Sullivan, K. D., Lewis, H. C., Hill, A. A., Pandey, A., Jackson, L. P., Cabral, J. M., Smith, K. P., Liggett, L. A., Gomez, E. B., Galbraith, M. D. et al. (2016). Trisomy 21 consistently activates the interferon response. *eLife* 5, e16220. 10.7554/eLife.16220 PMC5012864 27472900

[BIO062807C115] Sullivan, K. D., Evans, D., Pandey, A., Hraha, T. H., Smith, K. P., Markham, N., Rachubinski, A. L., Wolter-Warmerdam, K., Hickey, F., Espinosa, J. M. et al. (2017). Trisomy 21 causes changes in the circulating proteome indicative of chronic autoinflammation. *Sci. Rep.* 7, 14818. 10.1038/s41598-017-13858-3 PMC5665944 29093484

[BIO062807C116] Takata, M., Tanaka, H., Kimura, M., Nagahara, Y., Tanaka, K., Kawasaki, K., Seto, M., Tsuruma, K., Shimazawa, M. and Hara, H. (2013). Fasudil, a rho kinase inhibitor, limits motor neuron loss in experimental models of amyotrophic lateral sclerosis. *Br. J. Pharmacol.* 170, 341-351. 10.1111/bph.12277 PMC3834758 23763343

[BIO062807C117] Tang, Y.-C., Yuwen, H., Wang, K., Bruno, P. M., Bullock, K., Deik, A., Santaguida, S., Trakala, M., Pfau, S. J., Zhong, N. et al. (2017). Aneuploid cell survival relies upon sphingolipid homeostasis. *Cancer Res.* 77, 5272-5286. 10.1158/0008-5472.CAN-17-0049 PMC5772763 28775166

[BIO062807C118] Tarui, T., Im, K., Madan, N., Madankumar, R., Skotko, B. G., Schwartz, A., Sharr, C., Ralston, S. J., Kitano, R., Akiyama, S. et al. (2020). Quantitative MRI analyses of regional brain growth in living fetuses with Down syndrome. *Cereb. Cortex* 30, 382-390. 10.1093/cercor/bhz094 PMC7029684 31264685

[BIO062807C119] Tiberi, A., Montagni, E., Borgonovo, G., Coulomb, E., Restani, L., Mascaro, A. L. A., Capsoni, S. and Cattaneo, A. (2025). Microglia drive synaptic and functional connectivity deficits in the Ts65Dn mouse model of Down syndrome by affecting inhibition. *Acta Neuropathol. Commun.* 14, 21. 10.1186/s40478-025-02189-4 PMC12822298 41408325

[BIO062807C120] Tielemans, B., Llambrich, S., Seldeslachts, L., Cremer, J., Tsui, H. C., Jonckheere, A. C., Marain, N. F., Riedel, M., Wouters, J., Herzen, J. et al. (2025). Cardiopulmonary and immune alterations in the Ts65Dn mouse model of Down syndrome and modulation by epigallocatechin-3-gallate-enriched green tea extract. *Pharmaceutics* 17, 1366. 10.3390/pharmaceutics17111366 PMC12655474 41304705

[BIO062807C121] Tourneux, P., Chester, M., Grover, T. and Abman, S. H. (2008). Fasudil inhibits the myogenic response in the fetal pulmonary circulation. *Am. J. Physiol. Heart Circ. Physiol.* 295, H1505-H1513. 10.1152/ajpheart.00490.2008 PMC2593508 18676688

[BIO062807C122] Tuttle, K. D., Waugh, K. A., Araya, P., Minter, R., Orlicky, D. J., Ludwig, M., Andrysik, Z., Burchill, M. A., Tamburini, B. A. J., Sempeck, C. et al. (2020). JAK1 inhibition blocks lethal immune hypersensitivity in a mouse model of Down syndrome. *Cell Rep.* 33, 108407. 10.1016/j.celrep.2020.108407 PMC7727402 33207208

[BIO062807C123] Tweedie, D., Luo, W., Short, R. G., Brossi, A., Holloway, H. W., Li, Y., Yu, Q. S. and Greig, N. H. (2009). A cellular model of inflammation for identifying TNF-alpha synthesis inhibitors. *J. Neurosci. Methods* 183, 182-187. 10.1016/j.jneumeth.2009.06.034 PMC2756970 19583982

[BIO062807C124] Tweedie, D., Frankola, K. A., Luo, W., Li, Y. and Greig, N. H. (2011). Thalidomide analogues suppress lipopolysaccharide-induced synthesis of TNF-alpha and nitrite, an intermediate of nitric oxide, in a cellular model of inflammation. *Open Biochem. J.* 5, 37-44. 10.2174/1874091X01105010037 PMC3141331 21792375

[BIO062807C125] Valenti, D., Stagni, F., Emili, M., Guidi, S., Bartesaghi, R. and Vacca, R. A. (2021). Impaired brain mitochondrial bioenergetics in the Ts65Dn mouse model of Down syndrome is restored by neonatal treatment with the polyphenol 7,8-dihydroxyflavone. *Antioxidants (Basel)* 11, 62. 10.3390/antiox11010062 PMC8773005 35052567

[BIO062807C126] Velazquez, R., Ash, J. A., Powers, B. E., Kelley, C. M., Strawderman, M., Luscher, Z. I., Ginsberg, S. D., Mufson, E. J. and Strupp, B. J. (2013). Maternal choline supplementation improves spatial learning and adult hippocampal neurogenesis in the Ts65Dn mouse model of Down syndrome. *Neurobiol. Dis.* 58, 92-101. 10.1016/j.nbd.2013.04.016 PMC4029409 23643842

[BIO062807C127] Vuong, C. K., Weber, A., Seong, P., Matoba, N., Chen, Y. J., Peyer, J., Younesi, S., Salinda, A., Gomez, D., Rivas, G. et al. (2026). A single-cell multiomic analysis identifies molecular and gene-regulatory mechanisms dysregulated in developing Down syndrome neocortex. *Science* 392, eaea1259 10.1126/science.aea1259 PMC13225313 42024758

[BIO062807C128] Wang, Q., Song, L.-J., Ding, Z.-B., Chai, Z., Yu, J.-Z., Xiao, B.-G. and Ma, C.-G. (2022). Advantages of Rho-associated kinases and their inhibitor fasudil for the treatment of neurodegenerative diseases. *Neural Regen. Res.* 17, 2623-2631. 10.4103/1673-5374.335827 PMC9165373 35662192

[BIO062807C129] Watson, L. A. and Meharena, H. S. (2023). From neurodevelopment to neurodegeneration: utilizing human stem cell models to gain insight into Down syndrome. *Front. Genet.* 14, 1198129. 10.3389/fgene.2023.1198129 PMC10267712 37323671

[BIO062807C130] Waugh, K. A., Minter, R., Baxter, J., Chi, C., Galbraith, M. D., Tuttle, K. D., Eduthan, N. P., Kinning, K. T., Andrysik, Z., Araya, P. et al. (2023). Triplication of the interferon receptor locus contributes to hallmarks of Down syndrome in a mouse model. *Nat. Genet.* 55, 1034-1047. 10.1038/s41588-023-01399-7 PMC10260402 37277650

[BIO062807C131] Wolff, A. W., Bidner, H., Remane, Y., Zimmer, J., Aarsland, D., Rascol, O., Wyse, R. K., Hapfelmeier, A. and Lingor, P. (2024a). Protocol for a randomized, placebo-controlled, double-blind phase IIa study of the safety, tolerability, and symptomatic efficacy of the ROCK-inhibitor Fasudil in patients with Parkinson's disease (ROCK-PD). *Front. Aging Neurosci.* 16, 1308577. 10.3389/fnagi.2024.1308577 PMC10899319 38419648

[BIO062807C132] Wolff, A. W., Peine, J., Höfler, J., Zurek, G., Hemker, C. and Lingor, P. (2024b). SAFE-ROCK: a phase I trial of an oral application of the ROCK inhibitor fasudil to assess bioavailability, safety, and tolerability in healthy participants. *CNS Drugs* 38, 291-302. 10.1007/s40263-024-01070-7 PMC10980656 38416402

[BIO062807C133] Xiong, W., Li, R., Li, B., Wang, X., Wang, H., Sun, Y., Wang, X., Li, Y. and Ren, F. (2023). Nobiletin mitigates D-galactose-induced memory impairment via improving hippocampal neurogenesis in mice. *Nutrients* 15, 2228. 10.3390/nu15092228 PMC10181043 37432372

[BIO062807C134] Yang, Y.-J., Bu, L.-L., Shen, C., Ge, J.-J., He, S.-J., Yu, H.-L., Tang, Y.-L., Jue, Z., Sun, Y.-M., Yu, W.-B. et al. (2020). Fasudil promotes alpha-synuclein clearance in an AAV-mediated alpha-synuclein rat model of Parkinson's disease by autophagy activation. *J. Parkinsons Dis.* 10, 969-979. 10.3233/JPD-191909 32568105

[BIO062807C135] Ye, Q., Li, X., Gao, W., Gao, J., Zheng, L., Zhang, M., Yang, F. and Li, H. (2024). Role of Rho-associated kinases and their inhibitor fasudil in neurodegenerative diseases. *Front. Neurosci.* 18, 1481983. 10.3389/fnins.2024.1481983 PMC11613983 39628659

[BIO062807C136] Yun, H. J., Perez, J. D. R., Sosa, P., Valdés, J. A., Madan, N., Kitano, R., Akiyama, S., Skotko, B. G., Feldman, H. A., Bianchi, D. W. et al. (2021). Regional alterations in cortical sulcal depth in living fetuses with Down syndrome. *Cereb. Cortex* 31, 757-767. 10.1093/cercor/bhaa255 PMC7786357 32940649

[BIO062807C137] Zhao, J., Zhou, D., Guo, J., Ren, Z., Zhou, L., Wang, S., Xu, B. and Wang, R. (2006). Effect of fasudil hydrochloride, a protein kinase inhibitor, on cerebral vasospasm and delayed cerebral ischemic symptoms after aneurysmal subarachnoid hemorrhage. *Neurol. Med. Chir. (Tokyo)* 46, 421-428. 10.2176/nmc.46.421 16998274

[BIO062807C138] Zhou, Y., Zhou, B., Pache, L., Chang, M., Khodabakhshi, A. H., Tanaseichuk, O., Benner, C. and Chanda, S. K. (2019). Metascape provides a biologist-oriented resource for the analysis of systems-level datasets. *Nat. Commun.* 10, 1523. 10.1038/s41467-019-09234-6 PMC6447622 30944313

[BIO062807C139] Zmijewski, P. A., Gao, L. Y., Saxena, A. R., Chavannes, N. K., Hushmendy, S. F., Bhoiwala, D. L. and Crawford, D. R. (2015). Fish oil improves gene targets of Down syndrome in C57BL and BALB/c mice. *Nutr. Res.* 35, 440-448. 10.1016/j.nutres.2015.02.007 25799055

