# Supplementary material for: Fasudil induces anti-inflammatory transcriptomic changes and increased proliferation in human trisomy 21 neural progenitor cells
Source: Biol Open. 2026 Sep 8;15(9):bio062807. doi: 10.1242/bio.062807 (PMC13602368; doi:10.1242/bio.062807)
Supplement: Supplementary file 1 [file biolopen-15-062807-s1.pdf]

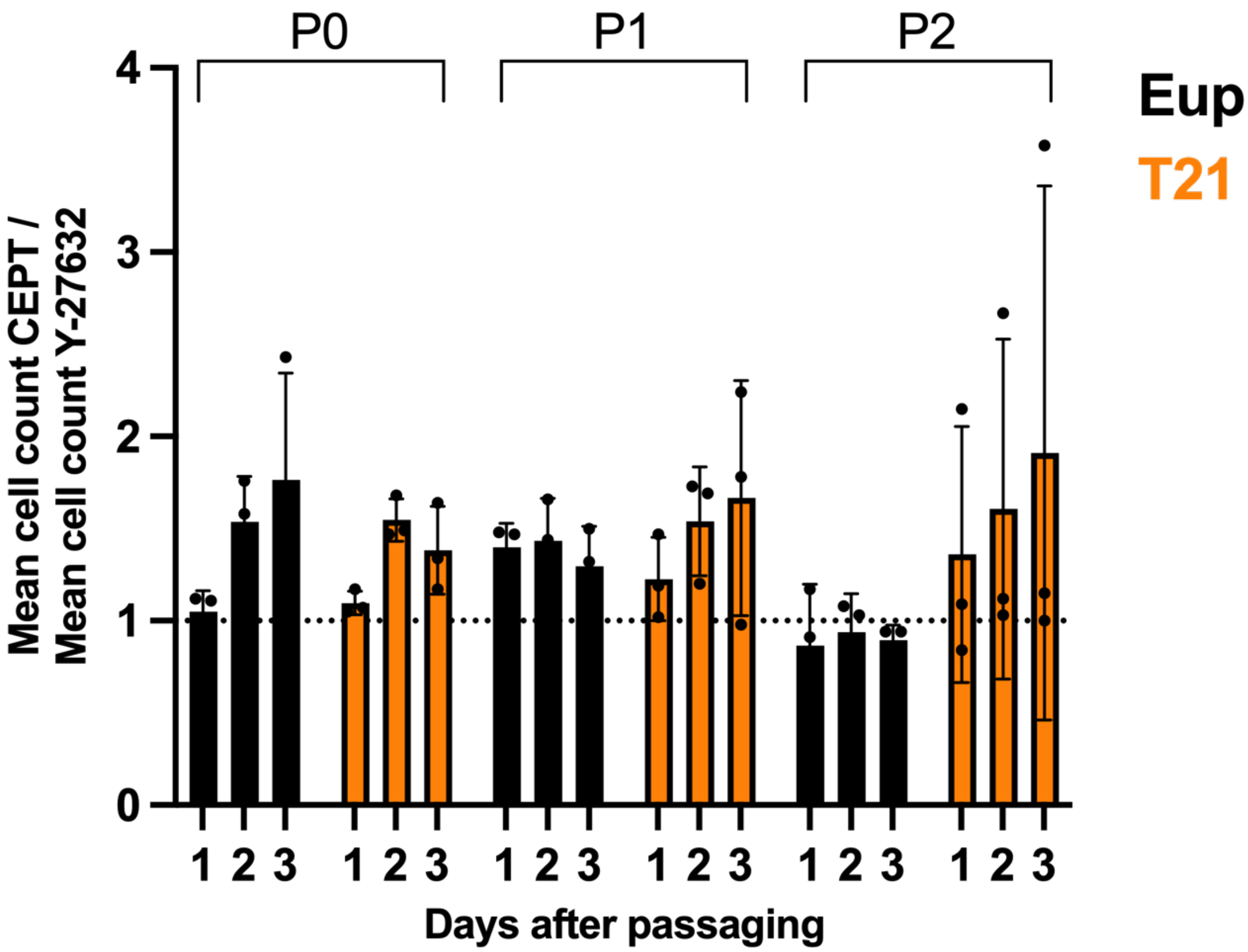

Fig. S1. Passaging with CEPT improved growth during neural induction of NPCs from iPSCs. More cells survived passaging with CEPT supplementation in comparison to Y-27632 supplementation in Eup and T21 lines. Ratios of CEPT-treated cell count to Y-27632 cell count in the same line are shown at one, two, and three days after passages 0, 1, and 2 (P0, P1, and P2, respectively). Ratio values >1 indicate greater cell numbers in CEPT-treated relative to Y-27632-treated lines. N = 3 Eup lines (EU6, EU7, EU12) and 3 T21 lines (TS3, TS4, TS8). Each data point = the mean of 3 replicates. Black = Eup lines, orange = T21 lines.

Biology Open • Supplementary information

Repeat 2

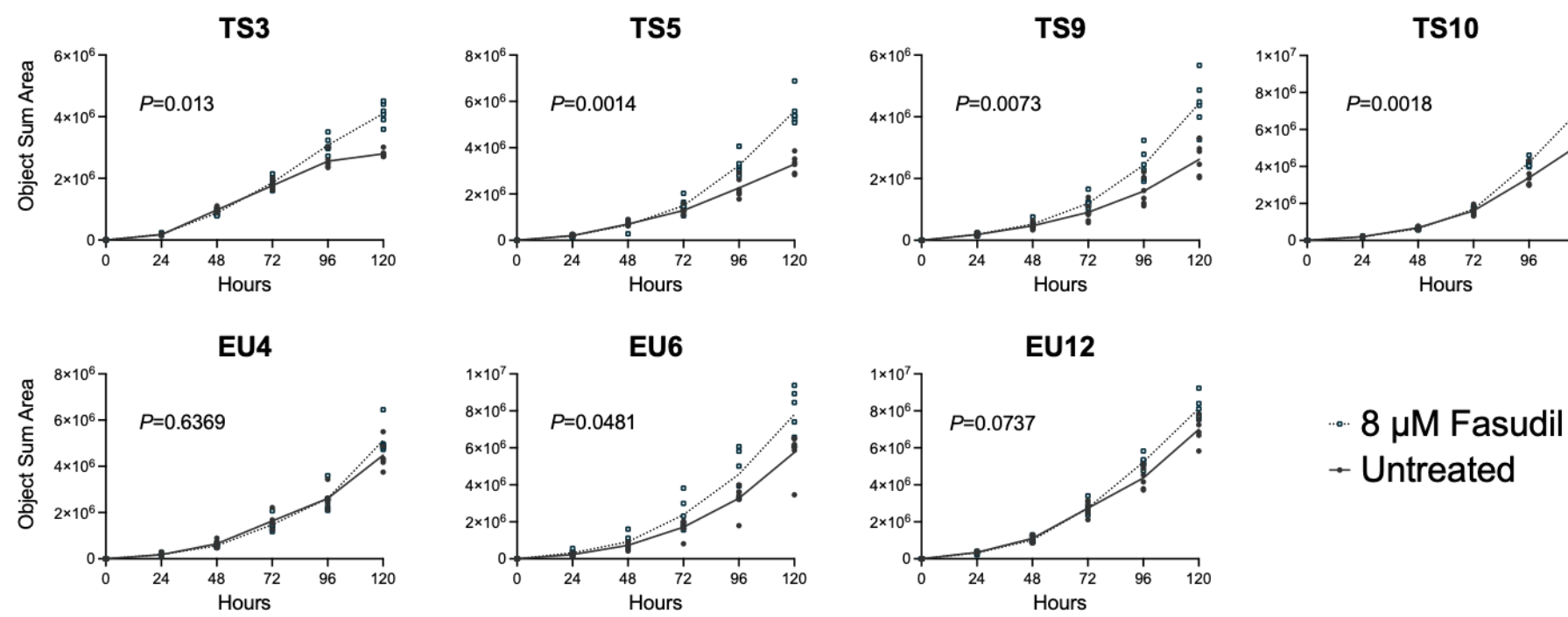

Repeat 3

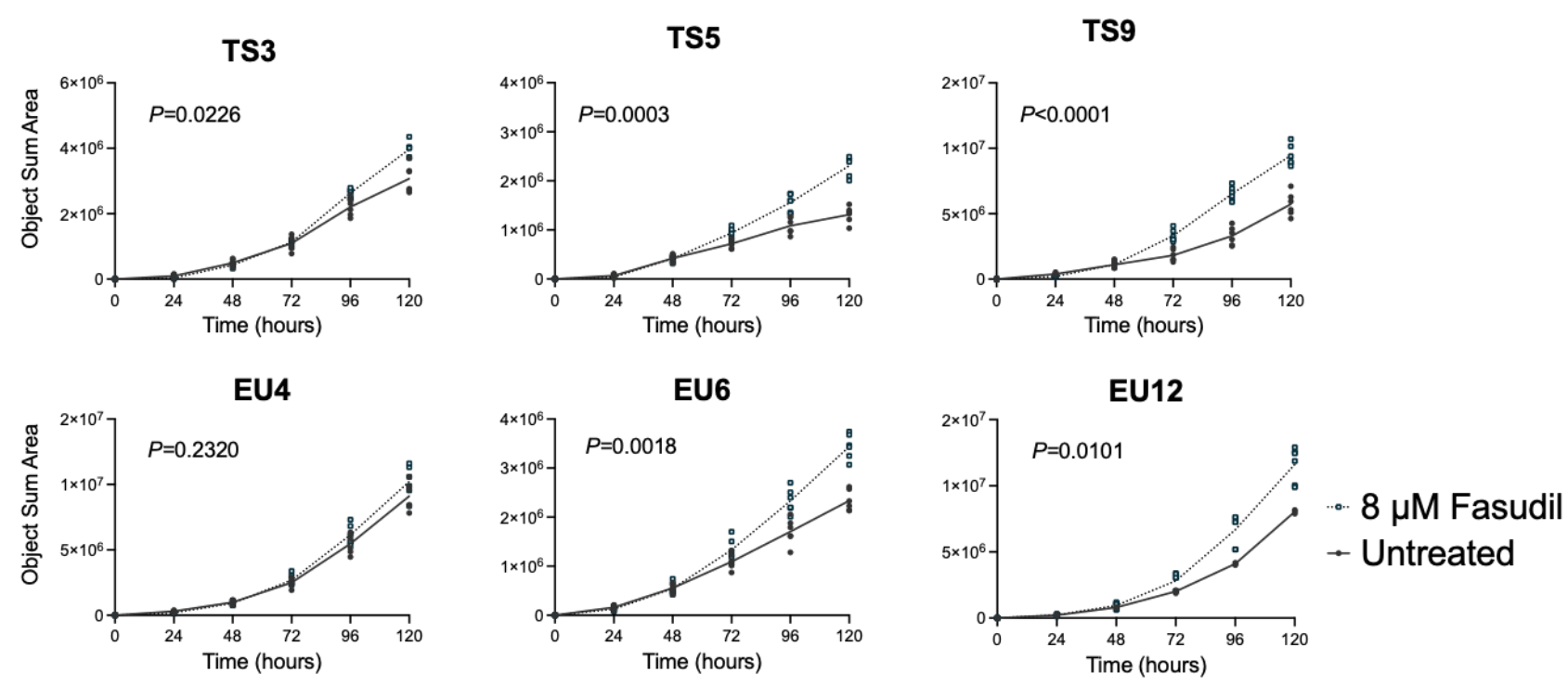

Fig. S2. Fasudil improves proliferation in T21 NPCs. Two out of three replicate experiments are shown; replicate three is shown in Fig. 1B. Increased growth consistently occurred following 8 µM fasudil treatment in all T21 lines and in 2 out of 3 Eup lines. Each graph shows a single, representative experiment for one cell line in the 6-well plate format experiments, with 6 replicate wells shown for each timepoint. Open squares = fasudil treated, closed circles = untreated. *P*-values are from 2-way ANOVA analysis. Data for all replicates are shown in Supp. Tab. S1b – S1d. T21 and Eup lines are named using the prefixes TS and EU, respectively, and these lines are described in Supp. Tab. S2.

Biology Open • Supplementary information

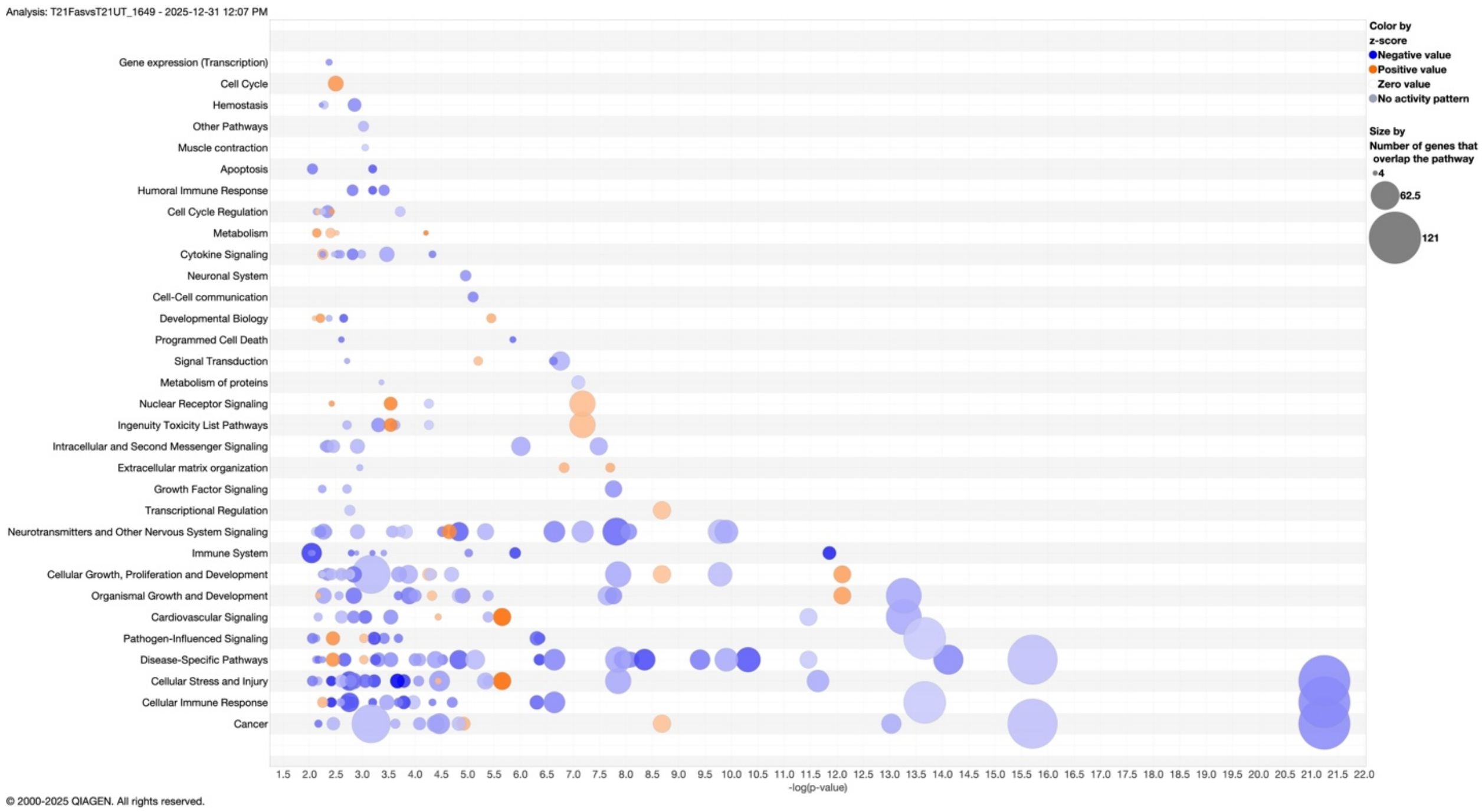

Fig. S3. Significantly enriched pathways in fasudil-treated T21 NPCs. Ingenuity Pathway Analysis for the 1649 T21 DEGs showed categories of significantly enriched pathways, including downregulation of numerous cellular immune response / immune system and cellular stress and injury pathways. Blue = negative Z score / pathway downregulation, and orange = positive Z score / pathway upregulation. Circle size represents the number of DEGs in the pathway.

Table S1. Candidate pharmacotherapies tested for effects on growth of euploid and T21 NPCs.

Available for download at
https://journals.biologists.com/bio/article-lookup/doi/10.1242/bio.062807#supplementary-data

Table S2. Euploid and T21 iPSCs and NPCs used in this study.

Available for download at
https://journals.biologists.com/bio/article-lookup/doi/10.1242/bio.062807#supplementary-data

Table S3. Viability and inflammatory marker results in LPS-challenged RAW 264.7 cells and IMG cells.

Available for download at
https://journals.biologists.com/bio/article-lookup/doi/10.1242/bio.062807#supplementary-data

Table S4. Comparison of gene expression in untreated T21 vs. untreated euploid NPCs.

Available for download at
https://journals.biologists.com/bio/article-lookup/doi/10.1242/bio.062807#supplementary-data

Table S5. Comparison of gene expression in fasudil-treated T21 vs. untreated T21 NPCs.

Available for download at
https://journals.biologists.com/bio/article-lookup/doi/10.1242/bio.062807#supplementary-data

Table S6. Identification of 1356 overlapping DEGs between T21Fas DEGs and T21 DEGs.

Available for download at
https://journals.biologists.com/bio/article-lookup/doi/10.1242/bio.062807#supplementary-data

Table S7. Comparison of gene expression in fasudil-treated euploid vs. untreated euploid NPCs.

Available for download at
https://journals.biologists.com/bio/article-lookup/doi/10.1242/bio.062807#supplementary-data

Table S8. Comparison of gene expression in EGCG-treated T21 vs. untreated T21 NPCs.

Available for download at
https://journals.biologists.com/bio/article-lookup/doi/10.1242/bio.062807#supplementary-data

Table S9. Comparison of gene expression in sphingosine-treated T21 vs. untreated T21 NPCs.

Available for download at
https://journals.biologists.com/bio/article-lookup/doi/10.1242/bio.062807#supplementary-data

Table S10. Comparison of gene expression in apigenin-treated T21 vs. untreated T21 NPCs and isoxsuprine-treated T21 vs. untreated T21 NPCs.

Available for download at
https://journals.biologists.com/bio/article-lookup/doi/10.1242/bio.062807#supplementary-data

Table S11. Comparison of gene expression in EGCG-treated, apigenin-treated, sphingosine-treated, and isoxuprine-treated euploid NPCs vs. untreated euploid NPCs.

Available for download at
https://journals.biologists.com/bio/article-lookup/doi/10.1242/bio.062807#supplementary-data
